# Resuscitation-associated endotheliopathy (RAsE): a conceptual framework based on a systematic review and meta-analysis

**DOI:** 10.1186/s13643-023-02385-0

**Published:** 2023-11-22

**Authors:** Nchafatso G. Obonyo, Declan P. Sela, Sainath Raman, Reema Rachakonda, Bailey Schneider, Louise E. See Hoe, Jonathon P. Fanning, Gianluigi Li Bassi, Kathryn Maitland, Jacky Y. Suen, John F. Fraser

**Affiliations:** 1 Critical Care Research Group, The Prince Charles Hospital, Brisbane, Australia; 2 Faculty of Medicine, The University of Queensland, Brisbane, Australia; 3 Initiative to Develop African Research Leaders (IDeAL), Kilifi, Kenya; 4 KEMRI-Wellcome Trust Research Programme, Kilifi, Kenya; 5 Wellcome Trust Centre for Global Health Research, Imperial College London, London, UK; 6 Institute of Molecular Bioscience, The University of Queensland, Brisbane, Australia; 7 Child Health Research Centre, The University of Queensland, Brisbane, QLD Australia; 8 Paediatric Intensive Care Unit, Queensland Children’s Hospital, South Brisbane, QLD Australia; 9 Division of Cardiac Surgery, Department of Surgery, Johns Hopkins School of Medicine, Baltimore, MD USA; 10 Nuffield Department of Population Health, University of Oxford, Oxford, UK; 11 Intensive Care Unit, St. Andrews War Memorial Hospital, Brisbane, QLD Australia; 12 Imperial College London, London, UK

**Keywords:** Shock, Resuscitation-associated endotheliopathy, Endothelial dysfunction, Microcirculation, Glycocalyx

## Abstract

**Introduction:**

Shock-induced endotheliopathy (SHINE), defined as a profound sympathoadrenal hyperactivation in shock states leading to endothelial activation, glycocalyx damage, and eventual compromise of end-organ perfusion, was first described in 2017. The aggressive resuscitation therapies utilised in treating shock states could potentially lead to further worsening endothelial activation and end-organ dysfunction.

**Objective:**

This study aimed to systematically review the literature on resuscitation-associated and resuscitation-induced endotheliopathy.

**Methods:**

A predetermined structured search of literature published over an 11-year and 6-month period (1 January 2011 to 31 July 2023) was performed in two indexed databases (PubMed/MEDLINE and Embase) per PRISMA guidelines. Inclusion was restricted to original studies published in English (or with English translation) reporting on endothelial dysfunction in critically ill human subjects undergoing resuscitation interventions. Reviews or studies conducted in animals were excluded. Qualitative synthesis of studies meeting the inclusion criteria was performed. Studies reporting comparable biomarkers of endothelial dysfunction post-resuscitation were included in the quantitative meta-analysis.

**Results:**

Thirty-two studies met the inclusion criteria and were included in the final qualitative synthesis. Most of these studies (47%) reported on a combination of mediators released from endothelial cells and biomarkers of glycocalyx breakdown, while only 22% reported on microvascular flow changes. Only ten individual studies were included in the quantitative meta-analysis based on the comparability of the parameters assessed. Eight studies measured syndecan-1, with a heterogeneity index, *I*^2^ = 75.85% (pooled effect size, mean = 0.27; 95% *CI* − 0.07 to 0.60; *p* = 0.12). Thrombomodulin was measured in four comparable studies (*I*^2^ = 78.93%; mean = 0.41; 95% *CI* − 0.10 to 0.92; *p* = 0.12). Three studies measured E-selectin (*I*^2^ = 50.29%; mean =  − 0.15; 95% *CI* − 0.64 to 0.33; *p* = 0.53), and only two were comparable for the microvascular flow index, MFI (*I*^2^ = 0%; mean =  − 0.80; 95% *CI* − 1.35 to − 0.26; *p* < 0.01).

**Conclusion:**

Resuscitation-associated endotheliopathy (RAsE) refers to worsening endothelial dysfunction resulting from acute resuscitative therapies administered in shock states. In the included studies, syndecan-1 had the highest frequency of assessment in the post-resuscitation period, and changes in concentrations showed a statistically significant effect of the resuscitation. There are inadequate data available in this area, and further research and standardisation of the ideal assessment and panel of biomarkers are urgently needed.

**Supplementary Information:**

The online version contains supplementary material available at 10.1186/s13643-023-02385-0.

## Introduction

Shock, defined as the clinical expression of circulatory failure, leads to a mismatch in the oxygen demand and delivery to tissue that is initially reversible [1]. However, progressive cellular and tissue hypoxia rapidly becomes irreversible [2], leading to a cascade of multi-organ failure and, ultimately, death if untreated [3]. Endothelial dysfunction is the putative underlying common pathway leading to this cascade. Johansson et al. recently described shock-induced endotheliopathy (SHINE), a profound sympathoadrenal hyperactivation in shock states that leads to endothelial stimulation, glycocalyx damage, and eventual compromise of end-organ perfusion [4].

The breakdown and shedding of the glycocalyx layer in shock, termed *endotheliopathy*, trigger a cascade of inflammatory and coagulation responses that can lead to *uncoupling* of the macro- and microcirculation [5]. Invasive arterial access for pressure monitoring and acute resuscitative interventions for shock, including venous lines for rapid volume expansion and fluid bolus administration, non-pulsatile blood flow (such as mechanical circulatory support with extracorporeal membrane oxygenation (ECMO), and other extracorporeal support therapies, such as renal replacement therapy (RRT), have traditionally targeted restoring well-validated macro-circulatory endpoints such as improving mean arterial blood pressure and urine output. Microcirculatory endpoints, however, have been difficult to quantify objectively, particularly in critical illness [6–8], yet recent data has shown that these strongly correlate with patient outcomes [9, 10].

Despite several direct and indirect techniques for assessing endothelial integrity and microcirculatory flow, they have yet to be standardised and adopted for evaluating microcirculation during acute resuscitation [11]. Therefore, inference on endothelial dysfunction is usually obtained from a singular or a combination of different techniques of microcirculatory assessment.

While SHINE provides a biological plausible concept for endotheliopathy observed in shock, it is incomplete and needs to be further expanded to include haemodynamic resuscitation. Resuscitative interventions may either cause endothelial dysfunction by themselves, augment the dysfunction initially instigated by shock, and may inhibit the natural defensive mechanisms that repair endothelial integrity. We coined the term *resuscitation-associated endotheliopathy* (*RAsE*) to encompass this phenomenon beyond SHINE.

Some literature supports this theory and has explored the mechanistic effects of aggressive resuscitation protocols, including rapidly administered fluid boluses for volume expansion [12]. In addition, increased circulating volume and pressure within blood vessels may generate shearing forces, leading to glycocalyx shedding and subsequent endothelial dysfunction [13, 14]. Therefore, it is plausible that aggressive volume-expansion resuscitation exacerbates endotheliopathy, predisposing to progressive worsening of end organs and ultimately adverse outcomes in the context of a shock state.

This comprehensive review aimed to systematically examine the literature for studies describing endothelial dysfunction following resuscitation therapies administered in shock states. We sought to synthesise recommendations for reporting standards in this fast-expanding research are by quantifying published literature.

## Methods

**Fig. 1:**
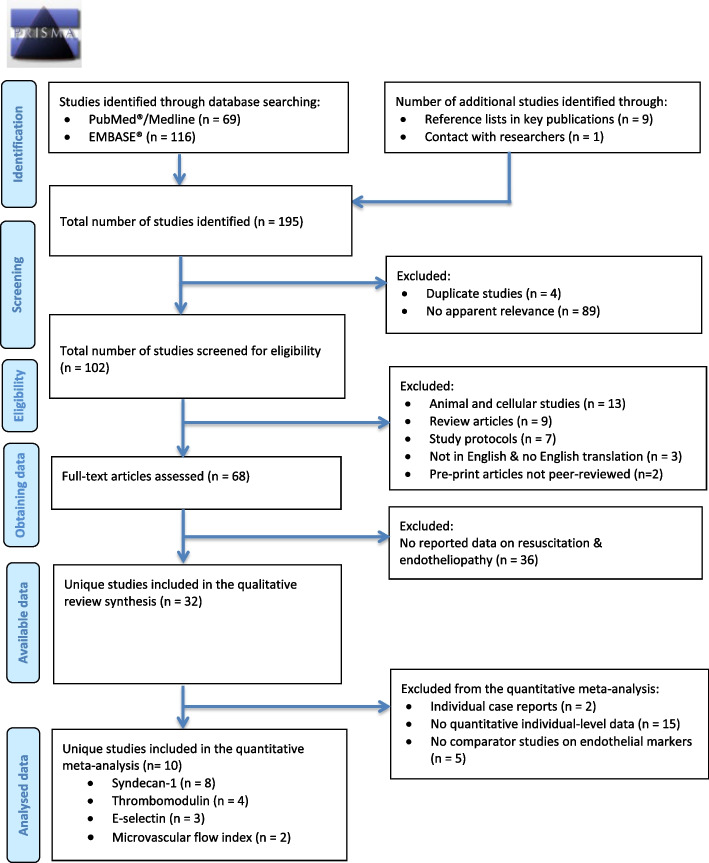
PRISMA flow diagram showing study selection, inclusion, and exclusion for the systematic review and meta-analysis

A predetermined systematic search, registered in the prospective international register of systematic reviews—PROSPERO (*ID: CRD42022349074*), was performed. Two online indexed medical databases, PubMed/MEDLINE and Excerpta Medica Database (Embase), were searched per the PRISMA guidelines [15] (Fig. 1).

All studies that reported on endothelial biomarkers data from critically ill humans who underwent resuscitation interventions were searched. The search terms used in [MeSH Terms] or [All Fields] were the keywords [‘resuscitation’] AND [‘endothelial dysfunction’ OR ‘endotheliopathy’ OR ‘endothelial damage’ OR ‘endothelial activation’]. The initial search was conducted from 1 January 2011 to 31 December 2021 and updated until 31 July 2023. A detailed description of the search strategy is included in Supplementary Table S1. All abstracts retrieved from the searches were filtered for duplicates, compiled in EndNote® (Thomson Reuters), and screened for relevance.

Eligible studies for inclusion were original clinical studies (including randomised controlled clinical trials, observational studies, case series, and case reports) published in English (or with an English translation). In these studies, the population was patients in shock states, and the intervention was administration of resuscitation fluid for treatment of the shock. Shock typically presents with a reduction in blood pressure, and most clinical guidelines recommend administration of volume expansion resuscitation to restore the microcirculatory parameters such as blood pressure and urine output. Therefore, some studies did not specify a comparator population, and it was assumed that all patients presenting in shock states were treated in accordance with the clinical resuscitation guidelines. The outcome of interest was a description of endotheliopathy following resuscitation for shock states by the following: (a) direct imaging for assessment of the microcirculatory function, measurement and quantification of (b) glycocalyx-breakdown biomarkers, and (c) mediators released from endothelial cells circulating in plasma. Relevant studies had their full manuscripts retrieved and reviewed by two independent reviewers in duplicate (NGO, DPS, assisted by RR, BS) (Supplementary Table S2). Assessment for risk of bias was performed based on the Cochrane risk of bias for randomised controlled trials [16] and the Newcastle–Ottawa scale (NOS) for observational studies [17] (Supplementary Table S3). Disagreements were resolved by consensus and additional senior review (SR, LESH). Reference lists and citations of the retrieved articles were also screened for relevance. The review articles and studies excluded did not describe endothelial dysfunction following resuscitation for circulatory shock or were conducted in animals.

### Data analysis

Meta-analysis of eligible studies presenting means and standard deviations with comparable microcirculatory and endothelial assessments was performed. Transformational analysis based on the method by Wan et al. [18] was performed for comparison of studies assessing similar markers of endothelial and microvascular dysfunction but reporting medians and interquartile ranges. A random-effects meta-analysis model was used, and all analysis was performed using STATA (ver. 17).

## Results

One-hundred and ninety-five articles were identified from the database searches, reference lists of key publications, and contact with authors. After an initial screening to remove duplicates and articles of no relevance, 102 studies were screened for eligibility, of which 68 full-text articles were accessed and reviewed. Thirty-two studies met the inclusion criteria and were included in the final qualitative synthesis as shown in Fig. 1. The details of these studies, including the population, intervention, control, and endothelial assessment, are presented in Table 1 below (additional details in Supplementary Table 2).

**Table 1:** Table showing population, intervention, control, and endothelial assessment of the included studies

Author and publication year	Study design	Population	Intervention	Number of participants (treatment and control groups)	Endothelial assessment		
					**Endothelial-glycocalyx breakdown biomarkers in plasma**	**Endothelial mediators in plasma**	**Microcirculation flow assessment**
**(1) Septic shock**							
Macdonald et al. (2023) [19]	Randomised controlled trial (biomarkers sub-study of the restricted fluid resuscitation in sepsis-associated hypotension, REFRESH, clinical trial)	Septic shock (sepsis-3 criteria) in adults over 18 years	Treatment (restricted fluid arm—early vasopressor and 250-mL intravenous fluid if *MAP* < 65 mmHg) *versus* control (usual care comprising initial fluid boluses 1000 mL and additional 500 mL, if required and later introduction of vasopressors)	95 patients randomised: restricted fluid arm (*n* = 49) *versus* controls/usual care (*n* = 46)	Syndecan-1 Syndecan-4 Hyaluronan heparan sulphate	ICAM VCAM VEGFR-1 E-selectin	-
Fernández-Sarmiento et al. (2023) [20]	Single-centre, prospective cohort study	Septic shock in children from 1 month to 18 years old	N/A; standard resuscitation protocol for management of haemodynamic instability in paediatric septic shock patients (20 mL/kg) comparing balanced or unbalanced crystalloids	106 patients observed (divided into two groups): unbalanced fluid (0.9% saline), *n* = 58, and balanced fluids (lactated Ringer’s, Hartmann or Plasma-Lyte 148 solution), *n* = 48	Syndecan-1	ANGPT2	GlycoCheck™ analysis of the perfused boundary region (PBR)
Saoraya et al. (2021a) [21]	Randomised controlled trial (post hoc analysis of the limited infusion rates on syndecan-1 Shedding, LIFE3S, clinical trial)	Septic shock (Sepsis-3 criteria) in adults over 18 years	Treatment (limited rate 10 mL/kg/h Ringer’s lactate) *versus* control (standard rate 30 mL/kg/h bolus or maximum rate of 2000 mL/h in the main LIFE3S trial)	95 participants (not divided into sub-groups in the post hoc analysis)	Syndecan-1	-	-
Saoraya et al. (2021b) [22]	Randomised controlled trial	Septic shock in adults ≥ 18 years	Initial bolus lactated Ringer’s solution Standard rate group (30 mL/kg/h, max. 2000 mL/h) vs limited-rate group (10 mL/kg/h)	96 patients randomised: standard-rate group (*n* = 48) versus limited-rate group (*n* = 48)	Syndecan-1	-	-
Hippensteel et al. (2019) [23]	Observational cohort study (based on patients in the Protocolized Care for Early Septic Shock, ProCESS, clinical trial)	Septic shock in adults ≥ 18 years		ProCESS cohort (*n* = 56)	Syndecan-1	sTM sFLT-1 tPA ANGPT2	-
Rovas et al. (2019) [24]	Prospective observational cross-sectional study	Septic shock in children and adults	Treatment standard resuscitation (Sepsis-3 criteria of septic shock) *versus* healthy controls	40 participants observed (divided into two groups): septic shock (*n* = 30) and healthy controls, adults only (*n* = 10)	Syndecan-1	-	GlycoCheck™ analysis of the perfused boundary region (PBR)
Wu et al. (2017) [25]	Prospective observational case–control study of post-operative patients	Septic shock in adults who had undergone open chest surgery	N/A; standard resuscitation protocol for septic shock patients	26 patients who had undergone thoracotomy: patients who had been admitted to ICU with severe sepsis post-op (*n* = 15) *versus* patients who had recovered (*n* = 11)	Syndecan-1	-	-
Bourcier et al. (2017) [26]	Prospective observational cohort study	Septic shock in patients aged 18 years or more	Standard resuscitation for septic shock using intravenous volume expansion and vasopressor treatment	37 participants observed (divided into two groups): with septic shock (*n* = 26) and without septic shock (*n* = 11)	-	-	Laser Doppler flowmetry
Meng et al. (2016) [27]	Non-blinded randomised controlled trial	Septic shock-induced ARDS in patients over 18 years	Treatment (early initiated continuous venovenous hemofiltration, ECVVH) *versus* control (non-ECVVH)	51 participants (divided into two-groups): ECVVH (*n* = 24) and non-ECVVH (*n* = 27)	-	sE-selectin	-
Müller et al. (2016) [28]	Observational cohort study (based on patients in the Scandinavian Starch for Severe Sepsis/Septic Shock, 6S, clinical trial)	Septic shock in patients over 18 years	Treatment (trial of hydroxyethyl starch 130/0.4, HES) *versus* control (Ringer’s acetate)	208 participants (divided into two groups): HES (*n* = 106) and Ringer’s acetate (*n* = 102)	Syndecan-1	sTM sCD40L Protein C tPA PAI-1	-
Katundu et al. (2016) [29]	Prospective observational cohort study	Septic shock in adults (no age specified) admitted to ICU and surviving to 7 days	N/a (no vitamin C was administered during the study period—instead, plasma sampling was done to assess vitamin C levels and correlate with other outcomes)	25 participants (i.e. 15 survivors and 10 non-survivors)	-	sVCAM-1 sE-selectin	-
**(2) Trauma and haemorrhagic shock**							
Peng et al. (2020) [30]	Observational cohort study (based on patients in the Fibrinogen in the initial Resuscitation of Severe Trauma, FiiRST, clinical trial)	Haemorrhagic shock in adult trauma patients over 18 years	Treatment (fibrinogen concentrate, FC 6 g) *versus* placebo (normal saline)	45 participants (divided into wo-groups for the sub-study): FC (*n* = 21) and placebo (*n* = 24)	Syndecan-1	sTM sE-selectin	-
Lopez et al. (2020) [31]	Prospective observational cohort study	Haemorrhagic shock in patients over 16 years	All participants received FFP treatment for haemorrhagic shock resuscitation	125 participants (divided into two groups for the analysis): normal ATIII (*n* = 50) *versus* ATIII deficient (*n* = 75)	Syndecan-1	ATIII	-
Welling et al. (2020) [32]	Retrospective observational cohort study	Endotheliopathy of trauma (EoT) and shock due to burns in patients over 16 years	Treatment volume replacement resuscitation for burns (i.e. using the modified Brooke formula 2 mL/kg/total body surface area) *versus* non-burn trauma controls (receiving volume replacement resuscitation of Ringer’s lactate and blood transfusion within 24 h)	458 participants (divided into two large groups): burn trauma (*n* = 68) and non-burn trauma (*n* = 390)	Syndecan-1	sTM	-
Gruen et al. (2020) [33]	Observational cohort study (based on patients in the Prehospital Air Medical Plasma, PAMPer, cluster randomised clinical trial)	Haemorrhagic shock in pre-hospital air medical transport patients aged between 18 and 90 years	Treatment (two units of plasma given pre-hospital and then standard resuscitation) *versus* controls (standard treatment for haemorrhagic shock during air medical transport)	405 participants (divided into two groups): treatment (*n* = 188) and controls (*n* = 217)	Syndecan-1	sTM VGEF	-
Naumann et al. (2019) [34]	Prospective observational cohort study (based on the observational pilot study of the effects of traumatic haemorrhagic shock and resuscitation on the microcirculation, MICROSHOCK study)	Traumatic injury and haemorrhagic shock in patients over 18 years	Observational study with no test treatment, all patients received the standard resuscitation for haemorrhagic shock	20 participants observed with no sub-groups	Syndecan-1	sTM	Incident dark field (IDF) assessment of microcirculatory flow
Naumann et al. (2018) [35]	Prospective observational cohort study (based on the Brain Biomarkers After Trauma Study, BBATS)	Haemorrhagic shock and endotheliopathy of trauma (EoT) in patients over 16 years	Pre-hospital tranexamic acid (TXA) for trauma patients	110 participants (divided into two large groups): trauma (*n* = 91) and non-trauma controls (*n* = 19)	Syndecan-1 (CD138)	sTM (CD141)	-
Gonzalez Rodriguez et al. (2018) [36]	Prospective observational cohort study	Endotheliopathy of trauma (EoT) in adults with traumatic brain injury (TBI) and polytrauma	Standard resuscitation for shock	360 participants (divided into two large groups): trauma (*n* = 331) and healthy controls (n = 29)	Syndecan-1	sTM	-
Stensballe et a. (2018) [37]	Randomised controlled trial (vasculopathic Injury and plasma as endothelial rescue-OCTAplasLG, VIPER-OCTA, trial)	Haemorrhagic shock in patients over 18 years	Treatment group (detergent-treated pooled plasma) *versus* control group (standard fresh frozen plasma, FFP)	44 participants (divided into two groups): OctaplastLG treatment (*n* = 23) and FFP controls (*n* = 21)	Syndecan-1	sTM sE-selectin sVE-cadherin	-
Turk et al. (2014) [38]	Randomised controlled trial	Endotheliopathy of trauma (EoT) in patients aged 18 years to 50 years with partial- or full-thickness burns covering 20–70% of the body surface area	Standard resuscitation and treatment for burns based on Parkland’s formula	60 participants (divided into two groups): burn patients (*n* = 30) and controls (*n* = 30)	-	-	Indirect assessment of the microcirculatory function using FMD after occlusion of the brachial artery
Tang et al. (2013) [39]	Prospective observational cohort study	Endotheliopathy of trauma (EoT) in patients older than 18-years	N/a (standard resuscitation practices for trauma patients)	82 participants (divided into two groups based on presence of coagulopathy): coagulopathy (*n* = 37) *versus* non-coagulopathy (*n* = 45)	-	von Willebrand factor (vWF) antigen	-
Junger et al. (2012) [40]	Randomised controlled trial (a priori sub-group analysis within the Resuscitation Outcomes Consortium, ROC, clinical trial)	Haemorrhagic shock in patients older than 15 years	(1) 7.5% hypertonic saline (HS) (2) 7.5% hypertonic saline + 6% dextran 70 (HSD) (3) 0.9% normal saline (control)	34 participants (divided into the three treatment groups): HS (*n* = 9) *versus* HSD (*n* = 8) *versus* control (*n* = 17)	-	sI-CAM-1 sV-CAM-1 sE-selectin sP-selectin	-
**(3) Cardiogenic shock**							
Meyer et al. (2020) [41]	Randomised controlled trial (endothelial dysfunction in resuscitated cardiac arrest, ENDO-RCA, sub-trial within the targeted temperature management, TTM, trial)	Cardiogenic shock in patients over 18 years with out-of-hospital cardiac arrest (OHCA), GCS < 8, and sustained ROSC for > 20 min	Treatment (iloprost infusion, 48 h of 1 ng/kg/min) *versus* placebo (0.9% saline infusion)	46 participants (divided into two groups for the ENDO-RCA sub-study): iloprost infusion (*n* = 13) and placebo (*n* = 33)	Syndecan-1	sTM sE-selectin, sVEGF, VEcad	-
Grand et al. (2020) [42]	Randomised controlled trial (ENDO-RCA, sub-trial within the TTM, trial)	Cardiogenic shock in patients over 18 years with out-of-hospital cardiac arrest (OHCA), GCS < 8, and sustained ROSC for > 20 min	Treatment (higher mean arterial pressure target of 72 mmHg, MAP 72) *versus* control (target mean arterial pressure of 65 mmHg, MAP65)	50 participants (divided into two groups for the sub-study): MAP72 (*n* = 24) and MAP65 (*n* = 26)	-	sTM	-
Ohbe et al. (2017) [43]	Prospective observational cohort study	Cardiogenic shock in patients aged over 20 years	Resuscitation for out-of-hospital cardiac arrest (R-OHCA)	28 participants observed (classified into two groups based on their 28-day survival): non-survivors (*n* = 13) and survivors (*n* = 21)	-	sTM vWF antigen	-
Bro-Jeppesen et al. (2017) [44]	Prospective observational cohort study (based on the TTM trial)	Cardiogenic shock in patients over 18 years with out-of-hospital cardiac arrest (OHCA), GCS < 8, and sustained ROSC for > 20 min	24-h target temperature management of either 33 °C (TTM33) or 36 °C (TTM36)	163 participants (divided into two groups for the analysis): TTM33 (*n* = 82) and TTM36 (*n* = 81)	Syndecan-1	sTM sE-selectin sVE-cadherin	-
Bro-Jeppesen et al. (2016) [45]	Prospective observational cohort study (based on the TTM trial)	Cardiogenic shock in patients over 18 years with out-of-hospital cardiac arrest (OHCA), GCS < 8, and sustained ROSC for > 20 min	24-h target temperature management of either 33 °C (TTM33) or 36 °C (TTM36)	163 participants (divided into two groups for the analysis): TTM33 (*n* = 82) and TTM36 (*n* = 81)	Syndecan-1	sTM sE-selectin sVE-cadherin	-
Omar et al. (2013) [46]	Prospective observational cohort study	Cardiogenic shock and septic shock in adults ≥ 18 years	N/a (standard resuscitation practices for cardiogenic and septic shock patients)	55 participants (divided into three groups): cardiogenic shock (*n* = 30), sepsis (*n* = 16), and controls (*n* = 9)	-	sE-selectin V-CAM I-CAM sVEGF	Microvascular flow index, MFI
**(4) Others**							
Monteiro et al. (2021) [47]	Prospective observational cohort study (based on the Randomised Evaluation of Sedation Titration for Respiratory Failure, RESTORE trial)	Acute respiratory failure (PALICC definition of ARDS) in patients aged 2 weeks to 17 years	Implementation of a nurse-implemented, goal-directed sedation protocol *versus* standard of care in the main RESTORE trial	432 participants (not divided into sub-groups in the post hoc analysis)	-	sTM	-
Case et al. (2020) [48]	Case report	Systemic capillary leak syndrome (SCLS) in a 63-year-old man who developed profound shock post-resuscitation	Crystalloid volume resuscitation	-	-	-	Systemic capillary leak and impaired microvascular endothelial function
Bøe et al. (2018) [49]	Case report	Systemic capillary leak syndrome (SCLS) in a 49-year-old woman with an upper respiratory tract infection who developed profound shock post-resuscitation	Crystalloid volume resuscitation	-	Syndecan-1 (CD138) and heparan sulphate	-	-
Somasetia et al. (2014) [50]	Randomised controlled trial	Endothelial dysfunction in dengue shock syndrome in children aged 2 years to 14 years	Treatment (hypertonic sodium lactate, HSL) *versus* control (Ringer’s lactate)	46 participants (divided into two groups): HSL (*n* = 24) and Ringer’s lactate (*n* = 22)	-	sVCAM-1	-

*PALICC*, Paediatric Acute Lung Injury Consensus Conference; *ARDS*, acute respiratory distress syndrome; *OHCA*, out-of-hospital cardiac arrest; *GCS*, Glasgow coma scale score; *ROSC*, return of spontaneous circulation; *MAP*, mean arterial pressure; *ATIII*, antithrombin III; *FFP*, fresh-frozen plasma; *EoT*, endotheliopathy of trauma; *ECVVH*, early-initiated continuous venovenous haemofiltration; *HES*, hydroxyethyl starch; *sTM*, soluble thrombomodulin; sE-selectin, soluble endothelial leucocyte adhesion molecule; *sVEGF*, soluble vascular endothelial growth factor; *sFLT-1*, soluble vascular endothelial growth factor receptor-1; *VEcad*, vascular endothelial cadherin; sVE-cadherin, soluble vascular endothelial cadherin; *vWF*, von Willebrand factor; *sCD40L*, soluble CD40 ligand; *tPA*, tissue-type plasminogen activator; *PAI-1*, plasminogen activator inhibitor-1; *sVCAM-1*, soluble vascular cell adhesion molecule-1; *FMD*, flow-mediated dilatation; *sV-CAM*, soluble vascular cell-adhesion molecule; *sI-CAM*, soluble intercellular adhesion molecule; *MFI*, microvascular flow index; sP-selectin, soluble platelet adhesion molecule; *ANGPT2*, angiopoietin 2

Of these thirty-two studies examining resuscitation and associated endotheliopathy included, there were 11 (34%) each on patients with trauma and haemorrhagic shock [19–29], and septic shock [30–40], and 6 (19%) on cardiogenic shock patients [41–46]. Two studies were case reports on systemic capillary leak syndrome (SCLS) [47, 48]. One study reported post-resuscitation endothelial dysfunction in acute respiratory failure [49] and another in dengue shock syndrome [50].

Twenty-four of the included studies (75%) were conducted in adults ≥ 18 years old (75%, i.e. 24/32) [20, 22, 23, 25–28, 30, 32–38, 40–48]. One study (3%) reported enrolment of a mix of adults and children [31].

### Endotheliopathy assessment

A combination of mediators released from endothelial cells and biomarkers of glycocalyx breakdown was reported in 47% [15/32] of studies [19–26, 34, 37, 39–41, 44, 45]. The number of studies reporting only endothelial cell mediators in plasma was 25% (8/32) [28, 29, 33, 35, 42, 43, 49, 50], while those that exclusively reported biomarkers of glycocalyx shedding were 12.5% (4/32) [30, 36, 38, 48] (Supplementary Table 2).

The microcirculatory flow was assessed in 22% (7/32) of the studies, 57% (4/7) of which used orthogonal polarisation spectroscopy (OPS) [23, 31, 46], with one study only reporting the perfused boundary region (PBR) rather than microvascular flow [39]. One study used laser Doppler flowmetry [32] and flow-mediated dilatation (FMD) [27], while another study did not clearly describe the technique used to evaluate microvascular flow [47].

### Meta-analysis

**Fig. 2:**
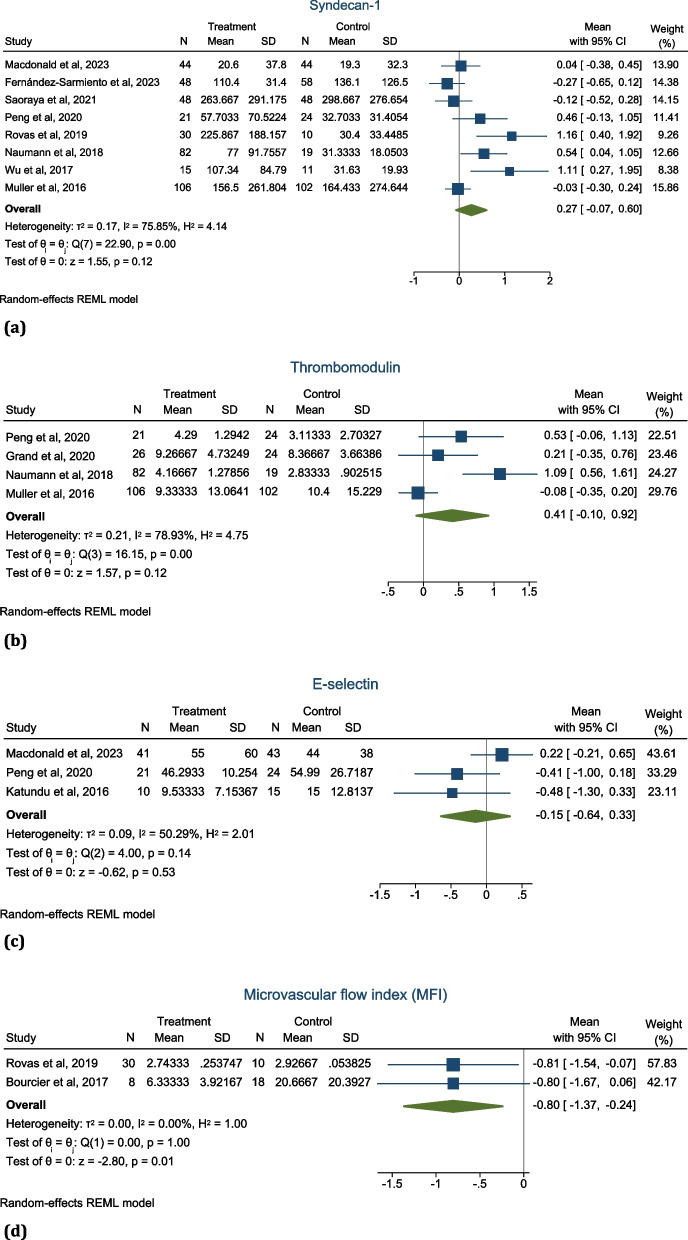
**a** Forest plot showing meta-analysis of eight studies that described syndecan-1 release post-resuscitation. Despite the high heterogeneity index, *I*^2^ = 75.87%, resuscitation caused a statistically significant release of syndecan-1 (pooled effect size; mean = 0.27; 95% *CI* - 0.07 to 0.60; *p* = 0.12). **b** Forest plot showing meta-analysis of four studies that described thrombomodulin release post-resuscitation. There was a high heterogeneity index between the studies with no significant effect of resuscitation on thrombomodulin release (*I*^2^ = 78.93%; mean = 0.41; 95% *CI* − 0.10 to 0.92; *p* = 0.12). **c** Forest plot of e-selectin release post-resuscitation. Only three studies described e-selectin release with high homogeneity but no significant effect of resuscitation on e-selectin release (*I*^2^ = 50.29%; mean =  − 0.15; 95% *CI* − 0.64 to 0.33; *p* = 0.53). **d** Forest plot of microvascular flow index (MFI) post-resuscitation. Only two-studies described MFI with high homogeneity but showed resuscitation significantly reduced the MFI (*I*.^2^ = 0%; mean =  − 0.80; 95% *CI* − 1.37 to − 0.24; *p* < 0.01)

Ten unique studies were included in the quantitative meta-analysis. However, it was only possible to analyse comparative endothelial assessments performed. For the glycocalyx biomarker syndecan-1, eight studies were included [20, 24, 31, 34, 36, 38–40], with a heterogeneity index *I*^2^ = 75.85% and pooled effect size mean = 0.27 (95% *CI* − 0.07 to 0.60; *p* = 0.12) (Fig. 2a). Four studies were included for the endothelial cell mediator thrombomodulin [20, 24, 34, 42], with a heterogeneity index *I*^2^ = 78.93% and pooled effect size mean = 0.41 (95% *CI* − 0.10 to 0.92; *p* = 0.12) (Fig. 2b). Comparable data was available for three studies for the endothelial cell mediator E-selectin [20, 35, 40] (*I*^2^ = 50.29%; mean =  − 0.15; 95% *CI* − 0.64 to 0.33; *p* = 0.53) (Fig. 2c) and only two studies for the microvascular flow index (MFI) [31, 32] (*I*^2^ = 0%; mean =  − 0.80; 95% *CI* − 1.37 to − 0.24; *p* < 0.01) (Fig. 2d). Graphical summaries of the meta-analyses and publication bias are presented as Funnel plots (Supplementary Fig. 1a–d) and Galbraith plots (Supplementary Fig. 2a–d), respectively.

## Discussion

Since the description of the SHINE phenomenon [4], this study systematically reviews post-resuscitation endotheliopathy in different types of circulatory shock. In summary, two-thirds of the studies included were published in 2017 or later, with equal numbers reporting on septic and haemorrhagic-trauma-related shock forming the bulk of the studies. While there are several biomarkers and techniques for assessing and quantifying endothelial and microcirculatory dysfunction, there are no standardised criteria for use in critically ill patients in shock. Only a few studies yielded comparable measurements for inclusion in the meta-analysis. Eight studies quantitatively compared syndecan-1, and four compared thrombomodulin that were included in the meta-analysis. However, these had relatively elevated heterogeneity indices indicative of underlying variability in the original studies. E-selectin had three comparable studies, while microvascular flow index (MFI) had only two comparable studies with high homogeneity. In the meta-analysis, only MFI reached the statistical threshold of significance. Four other studies meeting the inclusion criteria describing endothelial dysfunction post-resuscitation were not included in the meta-analysis as they did not fit within the framework of SHINE described by Johanssen et al. Two of these studies were case reports on systemic capillary leak syndrome: one on acute respiratory failure and another on dengue shock syndrome.

### Knowledge gap in endothelial biomarker research

While there are several biomarkers and techniques for assessing and quantifying endothelial and microcirculatory dysfunction, there are no standardised clinical criteria. Therefore, clinical assessment and quantification of endothelial dysfunction in critical illness and during resuscitation need a consistent approach. Despite the previous description of microvascular dysfunction in critical illness [13], only a few studies yielded comparable measurements for inclusion in the meta-analysis. Injury to the endothelium and shedding of the glycocalyx trigger the inflammatory-coagulopathy cascade leading to progressive microvascular dysfunction [13, 51–53]. Most of the studies included in this review used a combination of biomarkers, including glycocalyx breakdown products and mediators released from endothelial cells [19–26, 34, 37, 39–41, 44, 45]. Syndecan was the most described glycocalyx breakdown product in the reviewed studies. It is a transmembrane proteoglycan that undergoes cytokine-mediated release during inflammation [54], with levels circulating in plasma increasing during shock states [4]. Of the syndecans classified, syndecan-1 is the most common in shock-induced inflammation and has been extensively described in a recent literature review [55]. Our results highlight the variability in reporting practices. Future reporting guidelines need to be more prescriptive to enable progress in this field of research.

### Relationship between biomarkers and resuscitation practices

The typical clinical presentation of shock states is reduced blood pressure, indicative of impaired perfusion to match tissue requirements. Tissue hypoxia activates neutrophils in microvessels, and the subsequent neutrophil accumulation induces endothelial damage [56, 57].

Different shock aetiologies could impact the endothelium differently (Supplementary Fig. 3a–c). For instance, in septic shock, diverse pathogens may cause varied profiles of endotheliopathy [51]. Despite these differences, shock types share similar phenotypic features as the shock progresses, including sympatho-adrenal activation, catecholamine-induced glycocalyx damage, and pro-coagulant profile [4]. A recent review highlighted contradictions between basic, preclinical, and clinical studies on the significance of glycocalyx damage as a marker of vascular permeability [58].

**Fig. 3:**
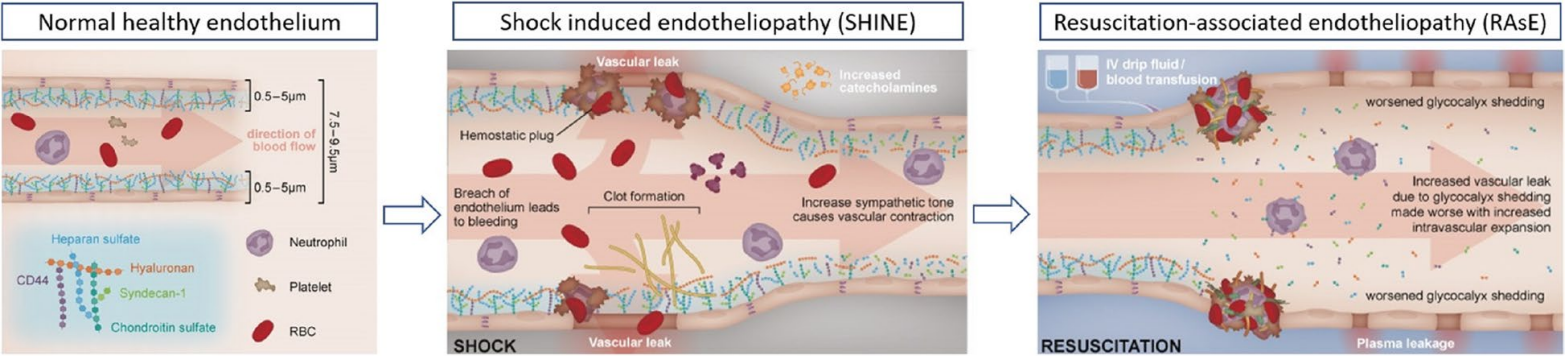
Illustration showing progression from normal healthy endothelium through shock-induced endotheliopathy (SHINE) following shock to resuscitation-associated endotheliopathy (RAsE) post-resuscitation. *RBC*, red blood cell; *IV*, intravenous

Figure 3 shows the hypothesised exacerbation of endotheliopathy during resuscitation for septic, haemorrhagic, and cardiogenic shock. Preclinical evidence has demonstrated that aggressive volume expansion in acute critical illness resuscitation leads to the progression and exacerbation of microcirculatory and endothelial dysfunction of endotoxaemic shock [12]. Based on this conceptual framework, it is plausible that initial damage to the endothelial-glycocalyx layer from the underlying shock could be further exacerbated by subsequent resuscitative interventions, thus predisposing to additional end-organ injury. Currently, there is limited clinical evidence for variation in endothelial injury following aggressive resuscitation for different shock states.

A summary of the pathophysiological mechanisms associated with the three different types of shock discussed are presented in Supplementary Fig. 3a–c. In septic shock, there is a relative reduction in the effective circulating volume due to vasodilatation and leakage into interstitial tissue. In contrast, in haemorrhagic shock, the decrease in the intravascular volume is due to blood loss. Volume replacement is the current standard of resuscitation for both these shock types [59–61]. In cardiogenic shock, global tissue hypoxia is secondary to poor perfusion, inducing a systemic inflammatory response syndrome (SIRS) comparable to sepsis [53, 62]. A similar SIRS response is seen with the initiation of extracorporeal membrane oxygenation (ECMO) [63]. The endothelial damage during shock and its subsequent exacerbation following resuscitation and reperfusion could have potential implications on clinical outcomes. It has been shown that disruption of the endothelial glycocalyx in cardiogenic shock is associated with worse patient outcomes [53, 64, 65]. Tsai et al. (2019) reported significantly higher levels of circulating vascular endothelial growth factor (VEGF), an endothelial survival factor associated with angiogenesis, at 72 h in patients who survived compared to those who died.

While higher resuscitation fluid volumes have been reported to correlate with higher levels of biomarkers such as syndecan-1 circulating in plasma [38], the clinical utility of syndecan-1 measurements during resuscitation remains limited. Thus, more research is required to correlate endothelial dysfunction biomarkers with the progression of end-organ dysfunction in shock states and during resuscitation [66]. Additionally, attainment of uniformity in analysing biomarkers of endotheliopathy requires some degree of standardisation of the time when they are measured since their relative abundance circulating in blood will vary over time. A proposed framework showing the major domains for the assessment and quantification of endotheliopathy in clinical studies is presented in Supplementary Table 4. With a better description of the endothelial injury biomarkers in the various resuscitation scenarios, investigators might be able to delineate the ‘epiphenomenon’ from real correlation and even risk factors.

This study has some limitations. One major limitation is the paucity of literature on resuscitation-associated endothelial dysfunction. Therefore, in order to understand which markers have been used clinically, studies were included on the basis of their reporting of endothelial markers post-resuscitation. Assessment of bias was performed in light of the fact that different study designs have been considered. As expected, the risk of bias was much lower in the randomised controlled studies than in the observational studies as reported in Supplementary Table 3. Funnel plots have been included to highlight the effects of smaller and non-randomised studies. Further description of the heterogeneity seen is provided by Galbraith plots that show the potential outliers mainly being non-randomised studies. However, the studies included in the meta-analysis are few, and more randomised studies investigating resuscitation-associated endothelial dysfunction are therefore required to address the knowledge gaps in this field.

Another limitation of this study was the lack of consistency in reporting of results in the studies that were reviewed. The lack of comparable biomarkers led to a reduction in the final number of studies that could be included in the quantitative meta-analysis resulting in high heterogeneity indices. Additionally, the application of transformations for the estimation of means and standard deviations from medians and interquartile ranges in the original publications were based on methods described by Wan et al. and are subject to mathematical assumptions [18]. Therefore, clearer reporting guidelines are necessary to achieve comparable and scientifically reproducible outcomes.

## Conclusion

In this review, we conceptualise the term resuscitation-associated endotheliopathy (RAsE) in relation to worsening endothelial dysfunction resulting from acute resuscitative therapies administered in shock states described as shock-induced endotheliopathy (SHINE). Unfortunately, there is neither consensus nor consistency in the definition of microvascular biomarkers in critically ill patients. Thus, additional research and standardisation of the ideal assessment and panel of biomarkers are urgently needed.

### Supplementary Information

**Additional file 1: Supplementary figures: S1. **Funnel plots**. S2. **Galbraith plots.** S3. **Shock pathophysiology.** Supplementary tables: S1.** Search terms.** S2.**All studies meeting the inclusion criteria. **S3. **Risk of bias assessment.** S4.**Framework for the assessment and quantification of endotheliopathy in clinical studies.

## Abbreviations

ARDS: Acute respiratory distress syndrome
ANGPT2: Angiopoietin 2
ATIII: Antithrombin III
ECMO: Extracorporeal membrane oxygenation
ECVVH: Early-initiated continuous venovenous haemofiltration
EoT: Endotheliopathy of trauma
FMD: Flow-mediated dilatation
FFP: Fresh-frozen plasma
GCS: Glasgow Coma Scale score
HES: Hydroxyethyl starch
MAP: Mean arterial pressure
MeSH: Medical Subject Headings
MFI: Microvascular flow index
NOS: Newcastle-Ottawa scale
OHCA: Out-of-hospital cardiac arrest
OPS: Orthogonal polarisation spectroscopy
PAI-1: Plasminogen activator inhibitor-1
PALICC: Paediatric Acute Lung Injury Consensus Conference
PBR: Perfused boundary region
RAsE: Resuscitation-associated endotheliopathy
ROSC: Return of spontaneous circulation
RRT: Renal replacement therapy
sCD40L: Soluble CD40 ligand
SCLS: Systemic capillary leak syndrome
sE-selectin: Soluble endothelial leucocyte adhesion molecule
sFLT-1: Soluble vascular endothelial growth factor receptor-1
SHINE: Shock-induced endotheliopathy
sI-CAM: Soluble intercellular adhesion molecule
SIRS: Systemic inflammatory response syndrome
sP-selectin: Soluble platelet adhesion molecule
sTM: Soluble thrombomodulin
sVCAM-1: Soluble vascular cell adhesion molecule-1
sVE-cadherin: Soluble vascular endothelial cadherin
sVEGF: Soluble vascular endothelial growth factor
Tpa: Tissue-type plasminogen activator
VEcad: Vascular endothelial cadherin
vWF: Von Willebrand factor
