# Supplementary material for: Resuscitation-associated endotheliopathy (RAsE): a conceptual framework based on a systematic review and meta-analysis
Source: Syst Rev. 2023 Nov 22;12:221. doi: 10.1186/s13643-023-02385-0 (PMC10664580; doi:10.1186/s13643-023-02385-0)
Supplement: Supplementary file 1 — Additional file 1: Supplementary figures: S1. Funnel plots. S2. Galbraith plots. S3. Shock pathophysiology. Supplementary tables: S1. Search terms. S2.All studies meeting the inclusion criteria. S3. Risk of bias assessment. S4.Framework for the assessment and quantification of endotheliopathy in clinical studies. [file 13643_2023_2385_MOESM1_ESM.docx]

**Supplementary Figures and Tables**

**(A) Supplementary Figures**

S1(a)

S1(b)


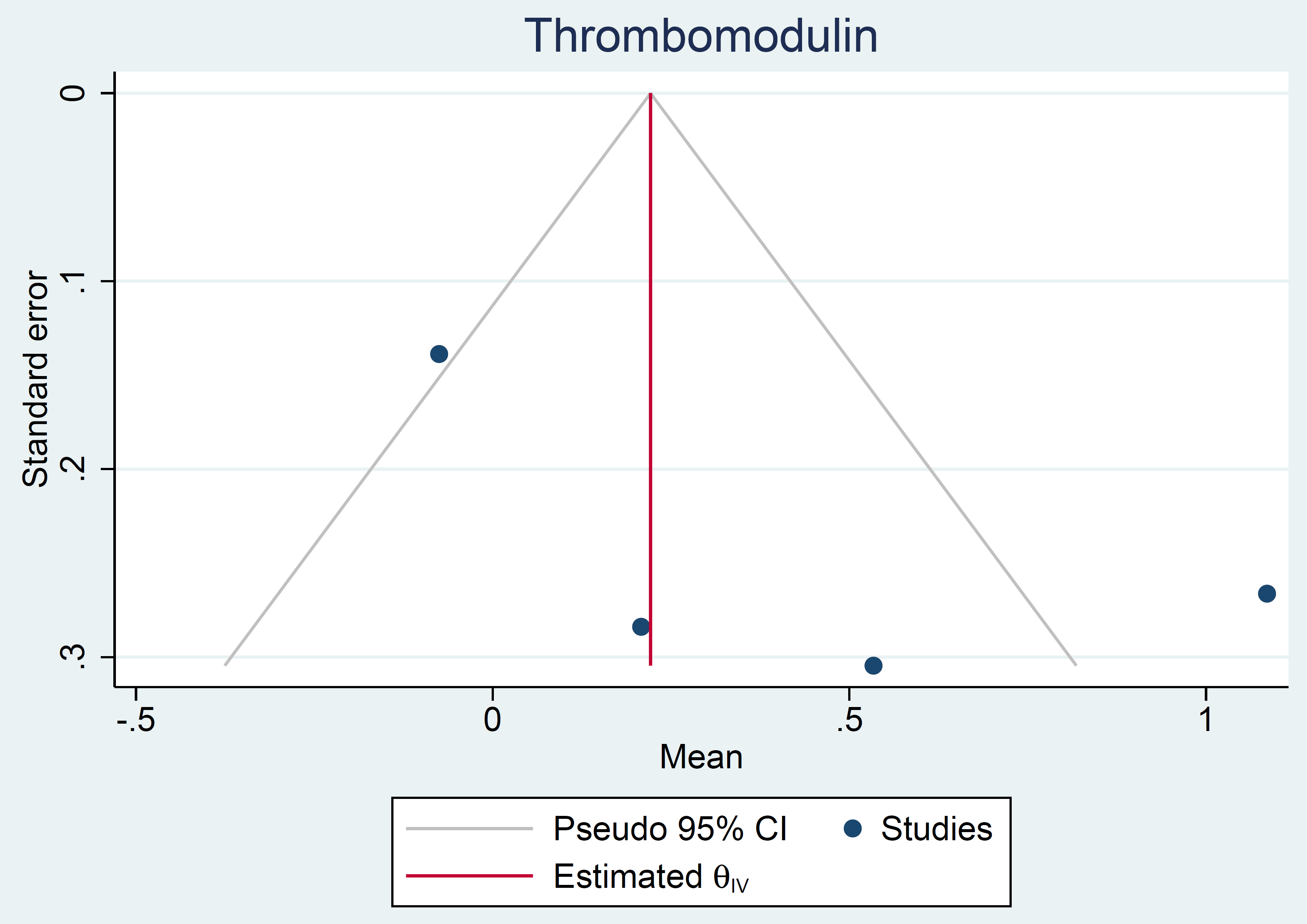


S1(c)

S1(d)


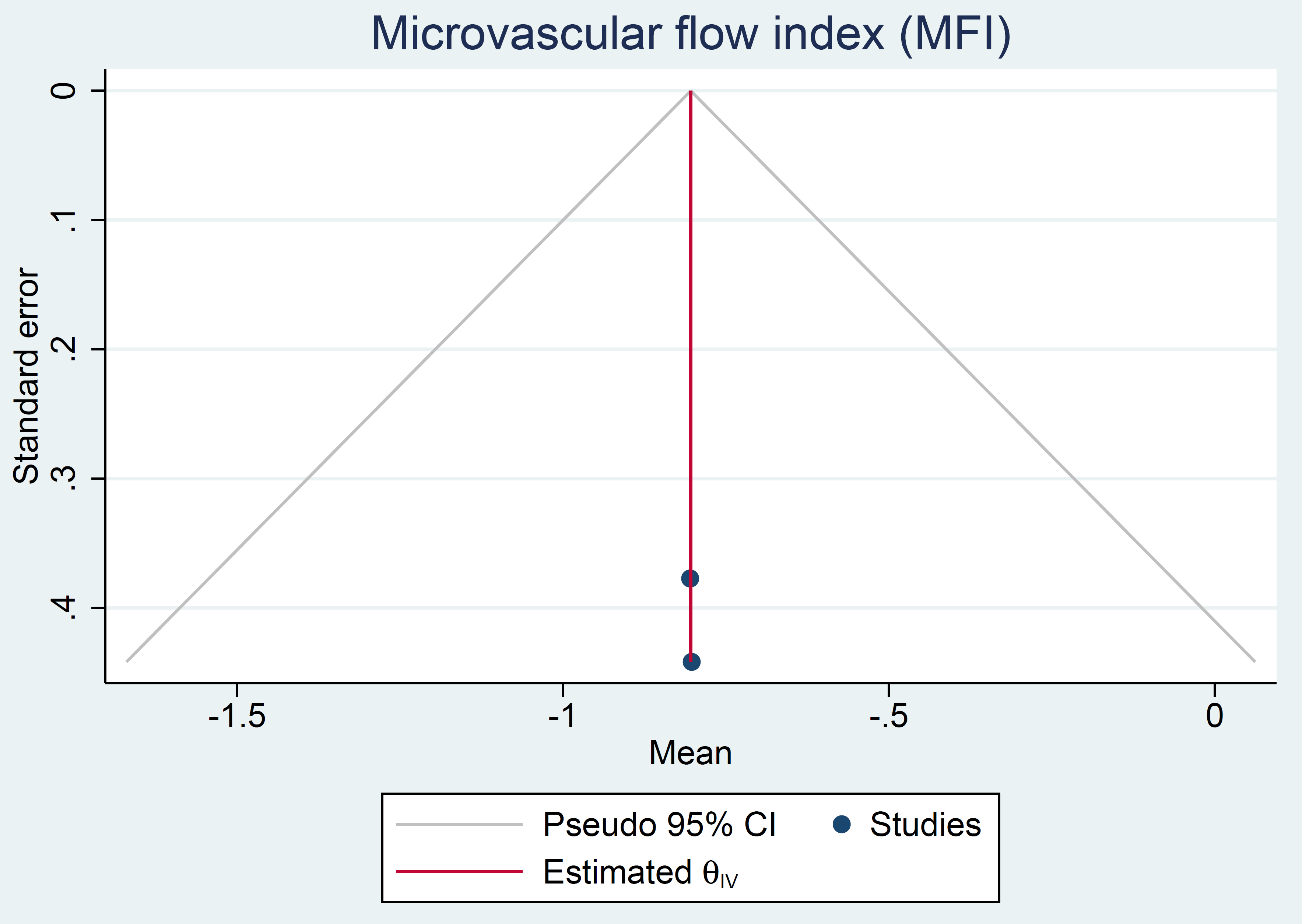


**Supplementary Figure S1**: Funnel plots of the meta-analysed studies plotted against the effect sizes reported for (a) syndecan-1, (b) thrombomodulin, (c) e-selectin and (d) microvascular flow index (MFI), respectively.

S2(a)

S2(b)


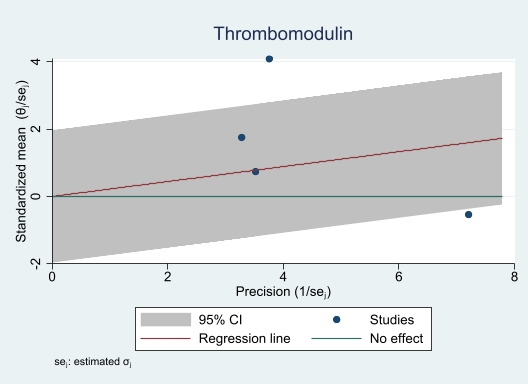


S2(c)

S2(d)


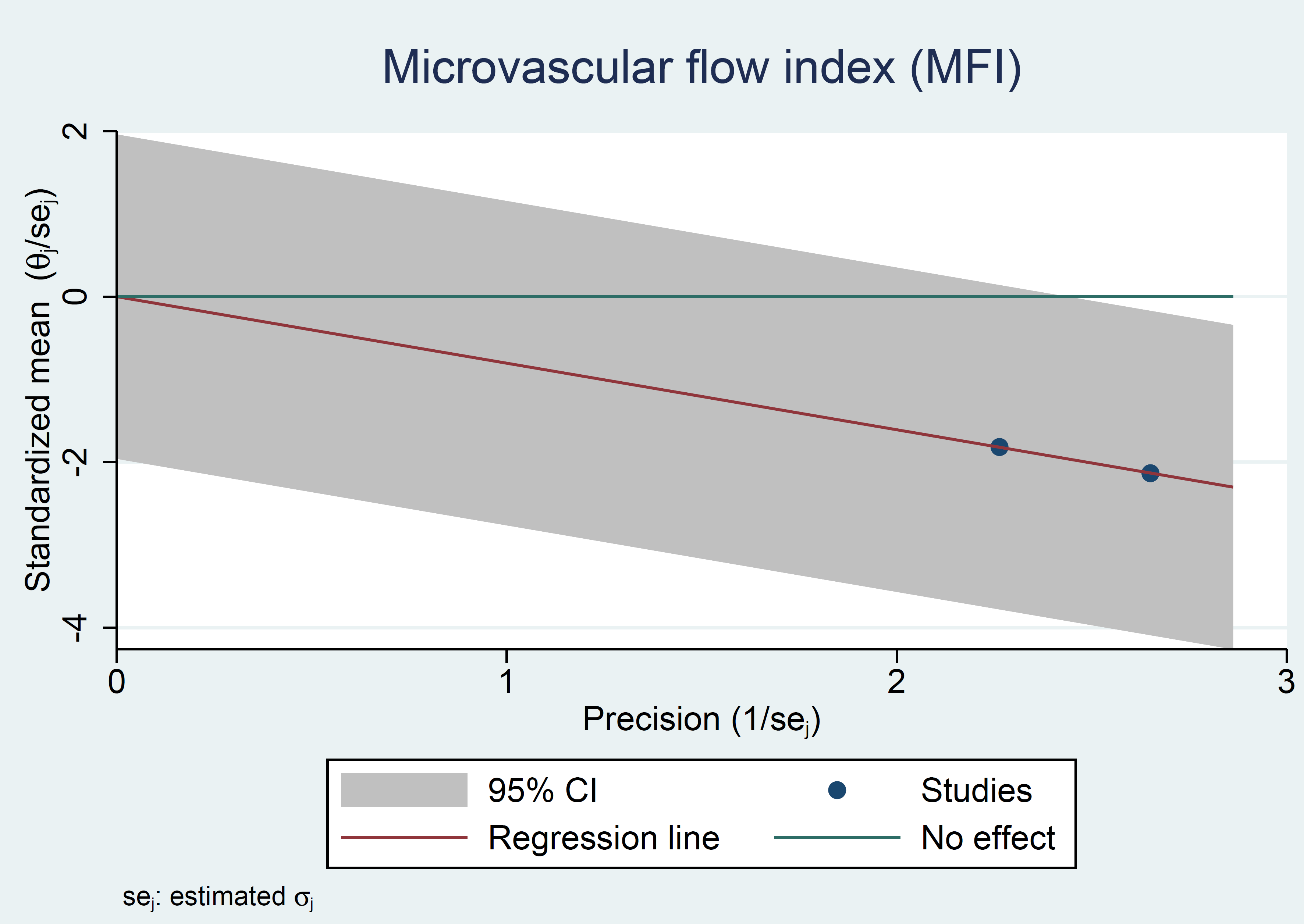


**Supplementary Figure S2**: Galbraith plots of the meta-analysed studies showing the effect sizes and potential outliers for (a) syndecan-1, (b) thrombomodulin, (c) e-selectin and (d) microvascular flow index (MFI), respectively.

**Supplementary Figure S3 (a-c)**: Schematic illustrations of the pathophysiology of shock

(a) Septic shock:

LPS & other Virulence Factors

Endothelium

Neutrophils

Monocytes

Infection

Cytokines e.g., TNF; IL-1; IL-6

Upregulation of Tissue Factor & PAI-1

Free Radicals e.g., Superoxide

Lipid Mediators e.g., Prostaglandins

Complement Cascade

Enhanced Coagulation

Chemotaxis; Cell Lysis

Thrombosis of Microvasculature

Vascular Instability

Coagulopathy, fever & vasodilation

Capillary Leak

Resuscitation

Sepsis + Multi-Organ Dysfunction

(b) Haemorrhagic Shock


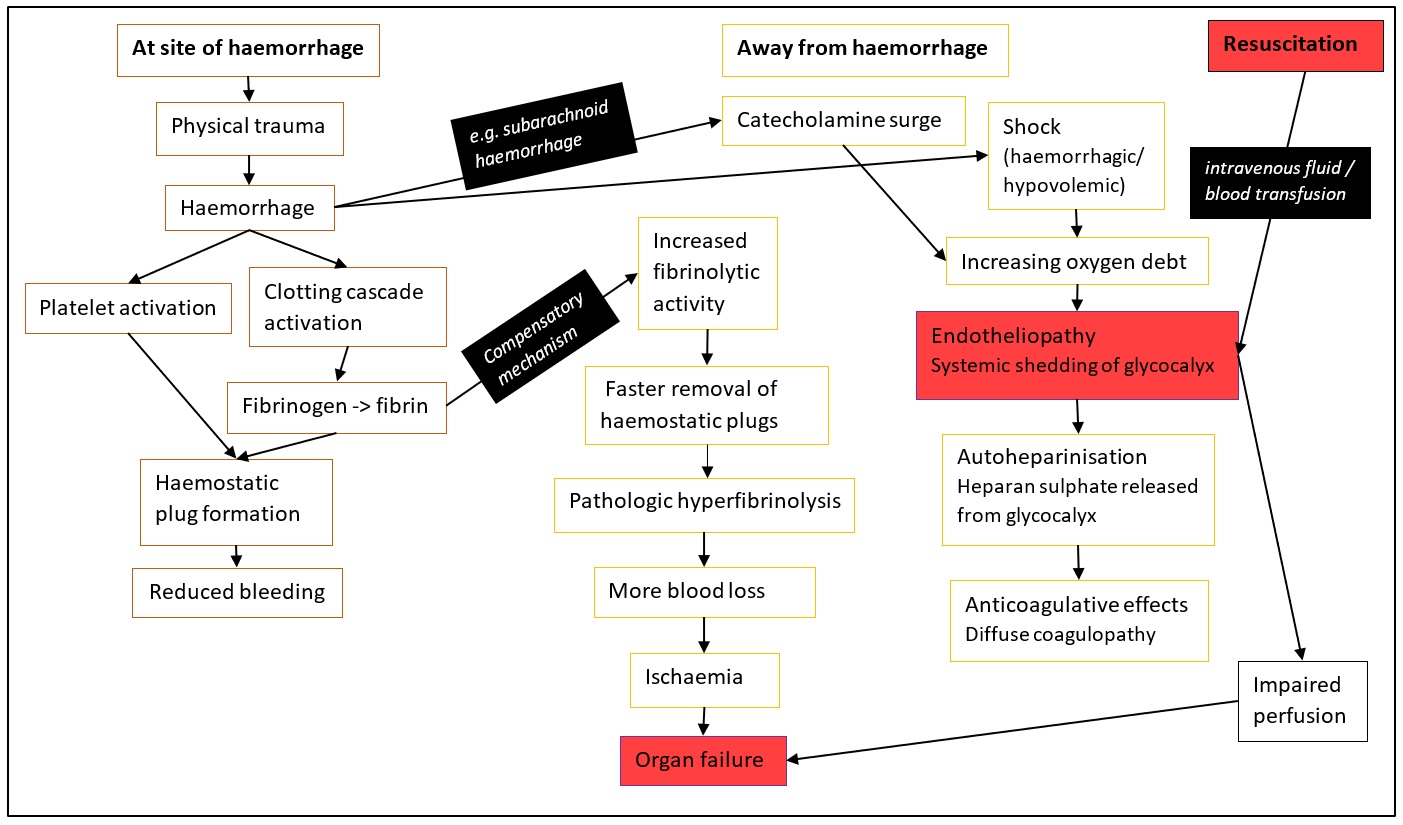


(c) Cardiogenic shock


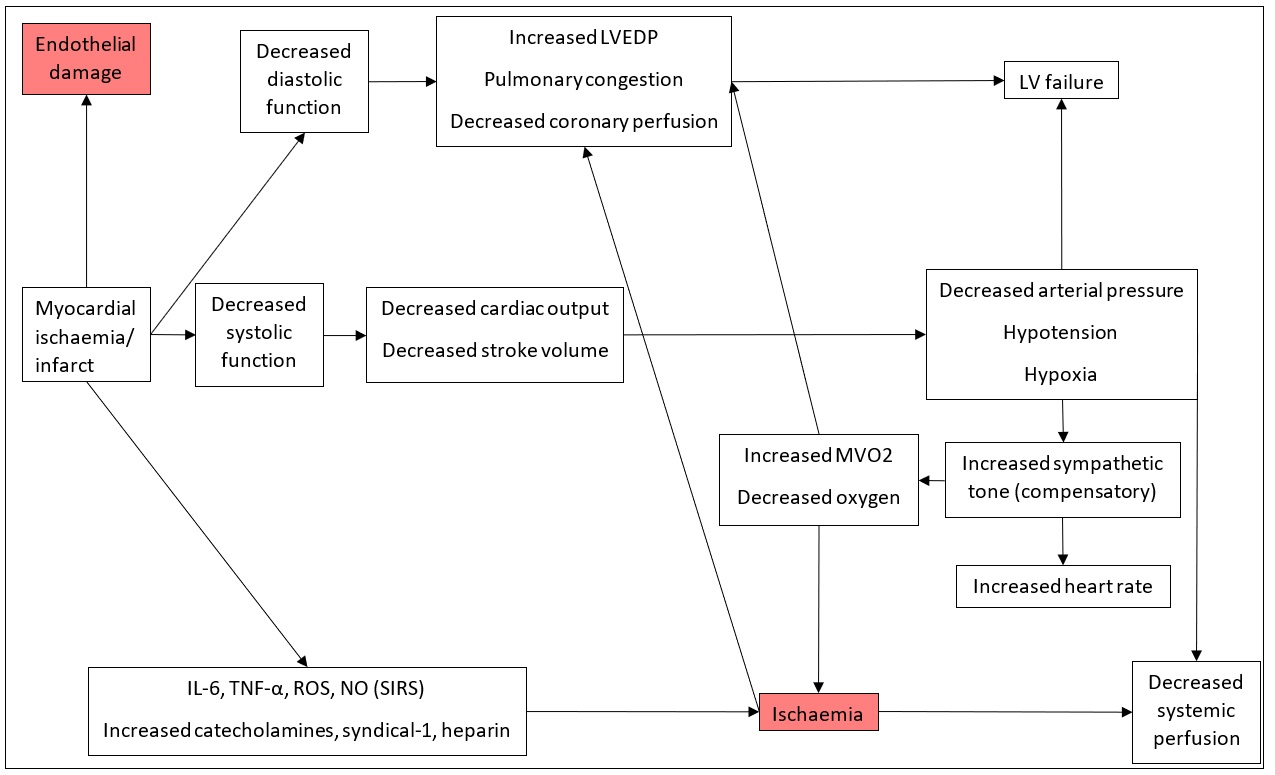


**(B) Supplementary Tables**

**Supplementary Table S1**: Search terms

| **Search fields** | **Search terms** |
| --- | --- |
| MeSH terms | ((resuscitation) AND ((endothelial dysfunction) OR (endothelial) AND (dysfunction))) OR ((resuscitation) AND (endotheliopathy)) OR ((resuscitation) AND (endothelial damage)) OR ((resuscitation) AND (endothelial activation)) |
| All Fields | ((resuscitation) AND ((endothelial dysfunction) OR (endothelial) AND (dysfunction))) OR ((resuscitation) AND (endotheliopathy)) OR ((resuscitation) AND (endothelial damage)) OR ((resuscitation) AND (endothelial activation)) |
| *MeSH, Medical Subject Headings* | |

**Supplementary Table S2**: Details of the included studies in the systematic review on resuscitation-associated endotheliopathy

| **Author & publication year** | **Population** | **Study design, dates and aims** | **Intervention** | **Number of participants (treatment and control groups)** | **Endothelial assessment** | | | **Principal findings** |
| --- | --- | --- | --- | --- | --- | --- | --- | --- |
|  |  |  |  |  | **Endothelial-glycocalyx breakdown biomarkers in plasma** | **Endothelial mediators in plasma** | **Microcirculation assessment** |  |
| **Septic shock** | | | | | | | | |
| Macdonald et al, 2023 ([19](#_ENREF_19)) | Septic shock (Sepsis-3 Criteria) in adults | Biomarkers sub-study of the REFRESH trial among adults (>18 years) presenting to the emergency department conducted between October 2016 and March 2018.  **Aim:** To test the hypothesis that intravenous fluid is a mediator of endothelial-glycocalyx shedding, endothelial cell activation and inflammation during the resuscitation phase of septic shock | Treatment (Restricted fluid arm - early vasopressor and 250mL intravenous fluid if MAP <65mmHg) *versus* control (usual care comprising initial fluid boluses 1000mL and additional 500mL, if required and later introduction of vasopressors) | 95 patients randomised: Restricted fluid arm (n=49) *versus* controls/usual care (n=46) | Syndecan-1  Syndecan-4  Hyaluronan  Heparan-sulphate | ICAM  VCAM  VEGFR-1  E-selectin | - | Significant differences over time between the groups were observed for hyaluronan (p=0.03) and syndecan-4 (p=0.01) but not for the other biomarkers.  The lack of a consistent signal across a range of the endothelial biomarkers and mediators assessed could be due to pre-existing endothelial-glycocalyx shedding by the time of randomisation or overlap between the fluid volumes administered in the two study groups. |
| Fernández-Sarmiento et al, 2023 ([20](#_ENREF_20)) | Septic shock in children from 1 month - 18 years old | Single centre, prospective cohort study in children with sepsis or septic shock requiring crystalloid fluid bolus resuscitation admitted in the paediatric intensive care unit from January to December 2021  **Aim:** To assess the disruption of endothelial glycocalyx integrity in children with sepsis receiving fluid resuscitation with either balanced or unbalanced crystalloids | N/A; standard resuscitation protocol for management of haemodynamic instability in paediatric septic shock patients (20mL/kg) comparing balanced or unbalanced crystalloids | 106 patients observed (divided into 2 groups); unbalanced fluid (0.9% saline), n = 58 and balanced fluids (lactated Ringer’s, Hartmann or Plasma-Lyte 148 solution), n = 48 | Syndecan-1 | ANGPT2 | GlycoCheck™ analysis of the perfused boundary region (PBR), an inverse parameter of the endothelial glycocalyx dimensions | Resuscitation with unbalanced crystalloid fluid (0.9% saline) led to worsening of endothelial-glycocalyx shedding among children with sepsis compared to those resuscitated with balanced solutions |
| Saoraya et al, 2021a ([21](#_ENREF_21)) | Septic shock (Sepsis-3 Criteria) in adults over 18-years | Post-hoc analysis of the Limited Infusion rates on Syndecan-1 Shedding (LIFE3S) RCT on fluid resuscitation in the emergency department conducted between November 2018 and February 2020.  **Aim:**  To explore the association of plasma syndecan-1 with the subsequent fluid requirements and clinical outcomes in Emergency Department patients with sepsis | Treatment (limited-rate 10ml/kg/h Ringer’s Lactate) *versus* control (standard-rate 30ml/kg/h bolus or maximum rate of 2000ml/h in the main LIFE3S trial) | 95 participants (not divided into sub-groups in the post-hoc analysis) | Syndecan-1 | - | - | Higher syndecan-1 levels correlated with higher 90-day mortality (p=0.02); higher SOFA scores (p<0.001); fluid administration; maximum dose of vasopressors required; and need for renal replacement therapy (p<0.05) |
| Saoraya et al, 2021b ([22](#_ENREF_22)) | Septic shock in adults ≥18 years | Prospective randomised controlled trial conducted between November 2018 and February 2020  **Aim:**  To investigate the effects of a limited infusion rate of fluid administered during the early phase of sepsis resuscitation on levels of plasma syndecan-1 compared to those of the standard fluid resuscitation rate. | Initial bolus lactated ringer’s solution:  Standard rate group (30mL/kg/hr, max. 2000mL/hr) vs limited-rate group (10mL/kg/hr) | 96 patients randomised: standard-rate group (n=48) versus limited-rate group (n=48) | Syndecan-1 | - | - | Primary outcome: There was no significant difference in change of syndecan-1 levels at 6 h compared to baseline between standard-rate and limited-rate groups.  Secondary outcomes: The treatment groups did not show any significantly different changes in 6-h lactate clearance, the proportion of patients with MAP ≥ 65 mmHg at 1 h and 6 h, or the P/F ratio at 6 h |
| Hippensteel et al, 2019 ([23](#_ENREF_23)) | Septic shock in adults ≥18 years | Prospective observational study, selecting patients enrolled in the ProCESS study (a multi-centre randomised controlled trial conducted across 31 hospitals in the USA, conducted between March 2008 to March 201)  **Aim:** To explore the potential importance of the glycocalyx  in human sepsis pathophysiology as well as the association between intravenous fluid resuscitation and glycocalyx degradation |  | ProCESS cohort (n=56) | Syndecan-1 | sTM  sFLT-1  tPA  ANGPT2 | - | There is an association between fluid resuscitation and glycocalyx degradation in sepsis |
| Rovas et al, 2019 ([24](#_ENREF_24)) | Septic shock in children and adults | Prospective, observational, cross-sectional study conducted between July 2017 and September 2017  **Aim:** To investigate the associations between dimensions of the endothelial-glycocalyx and established parameters of microcirculation dysfunction in sepsis | Treatment standard resuscitation (Sepsis-3 criteria of septic shock) *versus* healthy controls | 40 participants observed (divided into 2 groups); septic shock (n=30) and healthy controls, adults only (n=10) | Syndecan-1 | - | GlycoCheck™ analysis of the perfused boundary region (PBR), an inverse parameter of the endothelial glycocalyx dimensions and orthogonal polarisation imaging of microvascular flow using CapiScope side-stream dark field (SDF), and CytoCam incident dark field (IDF) | PBR was significantly higher in the septic patients compared to controls (p<0.0001), indicating damage to the glycocalyx.  Sublingual microcirculation was impaired in septic patients compared to controls as evidenced by a reduced microvascular flow index, MFI (p=0.002) and a lower proportion of perfused vessels, PPV (p=0.0004). |
| Wu et al, 2017 ([25](#_ENREF_25)) | Septic shock in adults who had undergone open chest surgery | Prospective observational single-centre study, conducted between January 2014 and May 2014  **Aim:** To determine the relationship between fluid resuscitation and glycocalyx degradation in patients with severe sepsis, and whether glycocalyx shedding has any prognostic value or necessitated changes in fluid resuscitation strategies in severe sepsis | N/A; standard resuscitation protocol for septic shock patients | 26 patients who had undergone thoracotomy: patients who had been admitted to ICU with severe sepsis post-op (n=15) *versus* patients who had recovered (n=11) | Syndecan-1 | - | - | Plasma syndecan-1 levels are significantly higher in patients with severe sepsis.  There is a correlation between plasma syndecan-1 levels and fluid balance for up to 48 hours.  The diagnostic value of lactate clearance for septic shock was higher than that of plasma syndecan-1 concentration |
| Bourcier et al, 2017 ([26](#_ENREF_26)) | Septic shock in patients aged 18-years or more | Prospective observational study  **Aim:** To assess microcirculatory and endothelia function in the skin | Standard resuscitation for septic shock using intravenous volume expansion and vasopressor treatment | 37 participants observed (divided into 2 groups); With septic shock (n=26) and without septic shock (n=11) | - | - | Laser Doppler flowmetry after transdermal iontophoresis of acetylcholine | Skin mottling in patients with septic shock is associated with regional endothelial dysfunction.  Following acetylcholine iontophoresis, increased blood flow to the skin was lower in patients with mottled skin (p<0.05) and in non-survivors (p<0.01) |
| Meng et al, 2016 ([27](#_ENREF_27)) | Septic shock-induced ARDS in patients over 18-years | Prospective single-centre non-blinded RCT conducted between November 2014 and March 2016.  **Aim:** To assess for change in endothelial function among patients with septic shock-induced ARDS and undergoing continuous venovenous haemofiltration (CVVH) | Treatment (early initiated continuous venovenous hemofiltration, ECVVH) *versus* control (non-ECVVH) | 51 participants (divided into 2-groups); ECVVH (n=24) and non-ECVVH (n=27) | - | sE-selectin | - | Treatment with ECVVH in addition to standard therapies improves endothelial function  (p<0.001), despite no difference in mortality between the treatment groups |
| Müller et al, 2016 ([28](#_ENREF_28)) | Septic shock in patients over 18-years | Sub-study of the Scandinavian Starch for Severe Sepsis/Septic Shock (6S trial) conducted between March 2009 and November 2011  **Aim:** To investigate the association between the resuscitation fluids and biomarkers of endothelial damage/coagulation impairment in septic shock patients | Treatment (trial of hydroxyethyl starch 130/0.4, HES) *versus* control (Ringer’s acetate) | 208 participants (divided into 2 groups); HES (n=106) and Ringer’s acetate (n=102) | Syndecan-1 | sTM,  sCD40L,  Protein C,  tPA,  PAI-1 | - | Increase in PAI-1 was associated with 90-day mortality (p<0.01). There was a decrease in endothelial damage following resuscitation with HES compared to Ringer acetate which was only significant for sTM (p=0.002).  However, there was higher overall mortality observed in the HES treatment group which may not be explained by early endothelial damage, thus endpoints based on surrogate markers could be misleading. |
| Katundu et al, 2016 ([29](#_ENREF_29)) | Septic shock in adults (no age specified) admitted to ICU and surviving to 7-days | Prospective observational study  **Aim:** To investigate whether vitamin C has effects on endothelial dysfunction, oxidative stress, hyperglycaemia, and patients’ outcomes in sepsis | n/a  (No vitamin C was administered during the study period – instead plasma sampling for was done to assess vitamin C levels and correlate with other outcomes) | 25 participants (i.e., 15 survivors and 10 non-survivors) | - | sVCAM-1,  sE-selectin | - | In this cohort of patients, the median vitamin C levels were lower than the normal reference ranges with no significant increase over the first 7-days (p=0.83).  Plasma sVCAM-1 levels were raised at admission and day-1 but had decreased significantly by day-7 (p<0.001). The baseline plasma sVCAM-1 levels were notably higher among non-survivors than survivors (p=0.01).  Plasma sE-selectin levels increased significantly from day-1 to day-7 (p=0.003), with higher baseline levels found in survivors than in the non-survivors (p=0.04). |
| **(2) Trauma and haemorrhagic shock** | | | | | | | | |
| Peng et al, 2020 ([30](#_ENREF_30)) | Haemorrhagic shock in adult trauma patients over 18-years | Retrospective evaluation of a sub-group in the Fibrinogen in the initial Resuscitation of severe trauma trial (FiiRST trial) conducted between October 2014 and November 2015.  **Aim:** To evaluate trauma induced coagulopathy (TIC), responses to early fibrinogen replacement therapy in trauma and to explore the molecular mechanism underlying TIC | Treatment (Fibrinogen concentrate, FC 6g) *versus* placebo (normal saline) | 45 participants (divided into 2-groups for the sub-study); FC (n=21) and placebo (n=24) | Syndecan-1 | sTM,  sE-selectin | - | Glycocalyx damage and endothelial cell activation were not evidenced by circulating levels of syndecan-1 and sE-selectin. Additionally, there was limited development of endotheliopathy observed by an elevation of circulating thrombomodulin levels post-resuscitation. |
| Lopez et al, 2020 ([31](#_ENREF_31)) | Haemorrhagic shock in patients over 16-years | Prospective, observational study in haemorrhagic shock patients conducted between August 2012 and November 2013.  **Aim:** To examine the effects of anti-thrombin III (ATIII) on endothelial expression of syndecan-1 and vascular barrier function in haemorrhagic shock resuscitated with fresh frozen plasma (FFP) | All participants received FFP treatment for haemorrhagic shock resuscitation | 125 participants (divided into 2-groups for the analysis); Normal ATIII (n=50) *versus* ATIII deficient (n=75) | Syndecan-1 | ATIII | - | There was no difference in the median syndecan-1 levels at admission, but on day 3, patients with ATIII deficiency displayed a significantly higher syndecan-1 levels than those with normal ATIII levels (p<0.01) suggestive of an association between ATIII deficiency and a higher degree of endothelial damage. |
| Welling et al, 2020 ([32](#_ENREF_32)) | Endotheliopathy of trauma (EoT) and shock due to burns in patients over 16-years | Retrospective analysis of observational data from a single-centre conducted between July 2012 and July 2017.  **Aim:** To investigate how endothelial injury due to burn trauma (EoT) and the shedding of the endothelial glycocalyx affect fluid resuscitation requirements and outcomes in burn patients | Treatment volume replacement resuscitation for burns (i.e., using the Modified Brooke Formula 2mL/kg/total body surface area) *versus* non-burn trauma controls (receiving volume replacement resuscitation of Ringer’s lactate and blood transfusion within 24-hours) | 458 participants (divided into 2 large groups); burn trauma (n=68) and non-burn trauma (n=390).  Participants in each large group were further subdivided into two groups based on the level of syndecan-1 being high or low (cut-off 40ng/mL) | Syndecan-1 | sTM | - | There were no significant differences between the burn *versus* non-burn trauma patients in levels of syndecan-1 (p=0.338) and sTM(p=0.925).  However, burn trauma sub-group of patients with higher syndecan-1 levels (n=14) had significantly higher levels of sTM and inhalation injury compared to those with lower syndecan-1 levels (n=54; p=0.012). Inhalational injury incidence could have been higher in these burn patients also due to an abundance of the glycocalyx in the lungs. |
| Gruen et al, 2020 ([33](#_ENREF_33)) | Haemorrhagic shock in pre-hospital air medical transport patients aged between 18 and 90 years | Post-hoc analysis of Prehospital Air Medical Plasma (PAMPer) cluster RCT conducted between May 2014 and September 2017.  **Aim:** To determine whether circulating markers of inflammation and endothelial damage are associated with pre-hospital plasma administration and clinical outcomes | Treatment (2-units of plasma given pre-hospital then standard resuscitation) *versus* controls (standard treatment for haemorrhagic shock during air medical transport) | 405 participants (divided into 2 groups); treatment (n=188) and controls (n=217)  Participants were further clustered based on type of trauma being either blunt (Cluster A) or penetrating (Cluster B) | Syndecan-1 | sTM,  VGEF | - | Blunt trauma (Cluster A) patients responded better to pre-hospital plasma compared to penetrating trauma (Cluster B). However, control group patients in Cluster A had a significantly lower 30-day survival compared to treatment group (p=0.016)    Markers of endothelial damage were significantly lower in the pre-hospital plasma group compared to the standard care group VEGF (p=0.0008), syndecan-1 (p=0.0047) and sTM (p=0.016).  As the severity of illness worsened, the concentration of endothelial biomarkers also increased in the control group but decreased in the plasma treatment group, suggesting that there may be a greater response to plasma in patients with greater injury severity. |
| Naumann et al, 2019 ([34](#_ENREF_34)) | Traumatic injury and haemorrhagic shock in patients over 18-years | Prospective observational sub-study within the MICROSHOCK longitudinal study conducted between July 2014 and June 2017  **Aim:** To investigate the relationship between plasma viscosity and sublingual microcirculatory flow | Observational study with no test treatment, all patients received the standard resuscitation for haemorrhagic shock | 20 participants observed with no sub-groups | Syndecan-1 | sTM | Incident dark field (IDF) assessment of microcirculatory flow dynamics | Lower volume of fluid administered correlated with higher plasma viscosity (p=0.0025).  Higher plasma viscosity was associated with a worse microvascular flow index, MFI (p=0.040), worse microcirculatory heterogeneity index, MHI (p=0.033) and worse point of care microcirculation, POE) (p=0.39), which persisted after adjusting for covariates (p=0.038, p=0.037 and p=0.044, respectively)  There were no associations between biomarkers of endothelial disruption (syndecan-1 and thrombomodulin) and plasma viscosity |
| Naumann et al, 2018 ([35](#_ENREF_35)) | Haemorrhagic shock and endotheliopathy of trauma (EoT) in patients over 16-years | Longitudinal prospective observational sub-study of patients in the Brain Biomarkers After Trauma Study (BBATS) conducted between May 2014 and February 2017  **Aim:** To investigate the association between EoT & multiple organ dysfunction syndrome (MODS) and whether tranexamic acid ameliorates EoT | Pre-hospital tranexamic acid (TXA) for trauma patients | 110 participants (divided into 2 large groups); trauma (n=91) and non-trauma controls (n=19)  Trauma patients were further subdivided into 2 groups; pre-hospital TXA (n=55) and no TXA (n=35) with one participant being excluded | Syndecan-1 (CD138) | sTM (CD141) | - | EoT occurs within minutes of injury.  MODS was associated with persistently raised biomarkers of EoT (P<0.05) but there were no differences in the levels of these biomarkers between the TXA and the non-TXA treatment groups. |
| Gonzalez Rodriguez et al, 2018 ([36](#_ENREF_36)) | Endotheliopathy of trauma (EoT) in adults with traumatic brain injury (TBI) and polytrauma | Prospective observational study conducted between July 2011 and May 2016  **Aim:** To determine the frequency of EoT after isolated and polytraumatic TBI and to investigate how glycocalyx breakdown affected outcomes in TBI patients | Standard resuscitation for shock | 360 participants (divided into 2 large groups); trauma (n=331) and healthy controls (n=29)  Trauma patients were further subdivided into 3 groups; isolated TBI (n=58), polytrauma and TBI (n=68), and non-TBI polytrauma (n=205) | Syndecan-1 | sTM | - | Patients with TBI combined with polytrauma had the highest levels of syndecan-1, followed by non-TBI polytrauma patients, then isolated TBI patients (p=0.0007).  Higher syndecan-1 levels were negatively associated with survival. Additionally, TBI with EoT patients had marked increases in levels of sTM.  TBI in trauma patients exacerbated breakdown of the glycocalyx leading to physiological derangements of the microcirculation and worse outcomes. |
| Stensballe et al, 2018 ([37](#_ENREF_37)) | Haemorrhagic shock in patients over 18-years | Investigator-initiated, single-center, blinded, randomized clinical pilot trial, (Vasculopathic Injury and Plasma as Endothelial Rescue-OCTAplasLG, VIPER-OCTA trial) conducted between November 2014 and December 2016  **Aim:** To compare the effect of coagulation support with solvent/detergent-treated  pooled plasma (OctaplasLG) versus standard fresh frozen plasma (FFP) on glycocalyx and endothelial  injury, bleeding, and transfusion requirements. | Treatment group (detergent-treated pooled plasma) *versus* control group (standard fresh frozen plasma, FFP) | 44 participants (divided into2 groups); OctaplastLG treatment (n=23) and FFP controls (n=21) | Syndecan-1 | sTM,  sE-selectin,  sVE-cadherin | - | Treatment with OctaplastLG reduced endothelial injury and glycocalyx shedding compared to standard FFP (p<0.05), however there was no difference in mortality between the two groups (p=0.76) |
| Turk et al, 2014 ([38](#_ENREF_38)) | Endotheliopathy of Trauma (EoT) in patients aged 18-years to 50-years with partial- or full-thickness burns covering 20%-70% of the body surface area. | Prospective randomised clinical study conducted between January 2010 and December 2011  **Aim:** To evaluate endothelial damage using flow mediated dilatation, FMD | Standard resuscitation and treatment for burns based on Parkland’s formula) | 60 participants (divided into 2 groups); burn patients (n=30) and controls (n=30) | - | - | Indirect assessment of the microcirculatory function using FMD after occlusion of the brachial artery | Despite resuscitation, burn patients had impaired FMD (p=0.037, day-1 vs 3-months), that also negatively correlated with high-sensitivity C-reactive protein (hs-CRP) on day-1 (p=0.02) and 3-months (p=0.04) after the burn.  There is endothelial dysfunction and low-grade inflammation in burn patients but the increase in FMD 3-months after the burn is attributable to effective treatment of the burn leading to gradual decrease in inflammatory mediators. |
| Tang et al, 2013 ([39](#_ENREF_39)) | Endotheliopathy of Trauma (EoT) in patients older than 18-years | Prospective observational study conducted between May and September 2012  **Aim:** To investigate the time course of platelet activation and endothelial dysfunction in trauma patients and to elucidate the relationship between coagulopathy and patient outcomes | n/a  (Standard resuscitation practices for trauma patients) | 82 participants (divided into 2 groups based on presence of coagulopathy); coagulopathy (n=37) *versus* non-coagulopathy (n=45) | - | Von Willebrand Factor (vWF) antigen | - | vWF antigen levels were significantly lower in patients with coagulopathy than in those without (p<0.05) and in non-survivors than in survivors (p<0.05).  Additionally, vWF antigen levels were significantly correlated with protein C and factor VII markers of coagulopathy (all p<0.05). |
| Junger et al, 2012 ([40](#_ENREF_40)) | Haemorrhagic shock in patients older than 15-years | *A priori* sub-group analysis of a larger randomised controlled trial under the Resuscitation Outcomes Consortium (ROC) conducted between May 2006 and August 2008  **Aim:** To determine how pre-treatment with hypertonic fluids affects the post-traumatic inflammatory response after haemorrhagic shock | (1) 7.5% hypertonic saline (HS)  (2) 7.5% hypertonic saline + 6% dextran-70 (HSD)  (3) 0.9% normal saline (control) | 34 participants (divided into the 3 treatment groups); HS (n=9) *versus* HSD (n=8) *versus* control (n=17) | - | sI-CAM-1,  sV-CAM-1,  sE-selectin,  sP-selectin, | - | HS used for initial resuscitation in traumatic haemorrhagic shock attenuated endothelial cell activation more than in the HSD group or in the controls receiving normal saline.  However, neither HS nor HSD reduced multi-organ dysfunction syndrome in post-trauma haemorrhagic shock patients, thus hypertonic resuscitation did not improve clinical outcome. |
| **(3) Cardiogenic shock** | | | | | | | | |
| Meyer et al, 2020 ([41](#_ENREF_41)) | Cardiogenic shock in patients over 18-years with out-of-hospital cardiac arrest (OHCA), GCS <8 and sustained ROSC for >20 min | Endothelial Dysfunction in Resuscitated Cardiac Arrest (ENDO-RCA), a sub-study of the Targeted Temperature Management (TTM) trial focusing on comatose OHCA patients conducted between February 2016 and February 2017.  **Aim**: To assess safety and efficacy of iloprost (a prostacyclin I2 analogue) administration on endothelial damage in OHCA patients | Treatment (iloprost infusion, 48-hours of 1ng/kg/min) *versus* placebo (0.9% saline infusion) | 46 participants (divided into 2-groups for the ENDO-RCA sub-study);  Iloprost infusion (n=13) and placebo (n=33) | Syndecan-1 | sTM,  sE-selectin, sVEGF, VEcad | - | At the 48-hour endpoint there was no difference in endothelial biomarkers between the treatment and placebo groups. However,  There was a rebound effect seen at 96-hours with increasing endothelial biomarkers after stopping iloprost infusion in the treatment group. |
| Grand et al, 2020 ([42](#_ENREF_42)) | Cardiogenic shock in patients over 18-years with out-of-hospital cardiac arrest (OHCA), GCS <8 and sustained ROSC for >20 min | Endothelial Dysfunction in Resuscitated Cardiac Arrest (ENDO-RCA), a sub-study of the Targeted Temperature Management (TTM) trial focusing on comatose OHCA patients conducted between February 2016 and February 2017.  **Aim**: To investigate the effect of a higher mean arterial blood pressure target (i.e., MAP72 *versus* MAP65) on biomarkers of organ injury | Treatment (higher mean arterial pressure target of 72mmHg, MAP 72) *versus* control (target mean arterial pressure of 65mmHg, MAP65) | 50 participants (divided into 2-groups for the sub-study); MAP72 (n=24) and MAP65 (n=26) | - | sTM | - | A MAP target of 72mmHg compared to 65mmHg did not lead to improved biomarkers of organ injury. |
| Ohbe et al, 2017 ([43](#_ENREF_43)) | Cardiogenic shock in patients aged over 20-years | Prospective observational study conducted between July 2011 and November 2013  **Aim:** To investigate whether a disintegrin-like and metalloprotease with thrombospondin type 1 motif 13 (ADAMTS13) is associated with neurologic outcome and mortality in patients with resuscitated out-of-hospital cardiac arrest (R-OHCA) and to correlate ADAMTS13 activity with endothelial damage in this cohort of patients | Resuscitation for out-of-hospital cardiac arrest (R-OHCA) | 28 participants observed (classified into 2 groups based on their 28-day survival);  non-survivors (n=13) and survivors (n=21) | - | sTM,  vWF antigen | - | Decreased plasma ADAMTS13 activity in patients with R-OHCA was associated with poor neurologic outcome (p=0.008), high mortality (p=0.02), higher inflammation (p<0.05), and worsened immune status (p=0.023).  There was a negative correlation between plasma ADAMTS13 activity with both sTM (R^2^=0.504 and p=0.021) and VWF antigen (R^2^=0.29 and p=0.021) |
| Bro-Jeppesen et al, 2017 ([44](#_ENREF_44)) | Cardiogenic shock in patients over 18-years with out-of-hospital cardiac arrest (OHCA), GCS <8 and sustained ROSC for >20 min | Post-hoc analysis of the Targeted Temperature Management (TTM) trial focusing on comatose OHCA patients conducted between February 2016 and February 2017.  **Aim**: To explore the haemodynamic effects of post-cardiac arrest syndrome (PCAS) and the associated endothelial activation & inflammatory response within the first 72-hours | 24-hours target temperature management of either 33^0^C (TTM33) or 36^0^C (TTM36) | 163 participants (divided into 2-groups for the analysis); TTM33 (n=82) and TTM36 (n=81) | Syndecan-1 | sTM,  sE-selectin,  sVE-cadherin | - | The TTM33 group of OHCA patients had higher levels of endothelial injury biomarkers which were associated with a higher heart rate in the first 24-hours and significantly higher vasopressor requirements at 48- and 72-hours when compared to the TTM36 group |
| Bro-Jeppesen et al, 2016 ([45](#_ENREF_45)) | Cardiogenic shock in patients over 18-years with out-of-hospital cardiac arrest (OHCA), GCS <8 and sustained ROSC for >20 min | Endothelial Dysfunction in Resuscitated Cardiac Arrest (ENDO-RCA), a sub-study involving post-hoc analysis of the Targeted Temperature Management (TTM) trial data focusing on comatose OHCA patients conducted between February 2016 and February 2017.  **Aim**: To investigate the effect of target temperature on endothelial damage and the prognostic value of endothelial damage on outcome in out-of-hospital cardiac arrest (OHCA) patients | 24-hours target temperature management of either 33^0^C (TTM33) or 36^0^C (TTM36) | 163 participants (divided into 2-groups for the analysis); TTM33 (n=82) and TTM36 (n=81) | Syndecan-1 | sTM,  sE-selectin,  sVE-cadherin | - | The TTM36 after OHCA was associated with lower endothelial activation, but not endothelial damage when compared to TTM33 |
| Omar et al, 2013 ([46](#_ENREF_46)) | Cardiogenic shock and septic shock in adults ≥18 years | Prospective observational single-centre study  **Aim:** To assess the association between microcirculatory dysfunction and inflammatory markers in the post-cardiac arrest state and to compare if microcirculatory dysfunction in post-cardiac arrest patients is similar to that seen in septic shock | n/a  (Standard resuscitation practices for cardiogenic and septic shock patients) | 55 participants (divided into 3 groups); cardiogenic shock (n=30), sepsis (n=16) and controls (n=9)  Cardiogenic shock patients were further sub-divided into in-hospital cardiac arrest (n=17) and out-of-hospital cardiac arrest (n=13) while 7 of the 16 sepsis patients had septic shock | - | sE-selectin,  V-CAM,  I-CAM,  sVEGF | Microvascular flow index, MFI | Microcirculatory dysfunction occurs early in post-cardiac arrest patients and restoration of microcirculatory function at 24-hours is associated with a good neurological outcome.  After adjustment for initial illness severity, post-cardiac arrest patients had a significantly lower MFI at 6-hours compared to sepsis patients. There was no correlation between MFI at 6h or 24h with E-selectin, v-CAM, or i-CAM in all the patients |
| **(4) Others** | | | | | | | | |
| Monteiro et al, 2021 ([47](#_ENREF_47)) | Acute respiratory failure (PALICC definition of ARDS) in patients aged 2 weeks to 17 years | Prospective observational study (post-hoc analysis of the multicentre clinical trial, Randomised Evaluation of Sedation Titration for Respiratory Failure, RESTORE trial conducted between June 2009 and December 2013).  **Aim:** To correlate levels of soluble thrombomodulin in children with acute respiratory failure with inflammation and vascular injury leading to multi-organ failure | Implementation of a nurse-implemented, goal-directed sedation protocol *versus* standard of care in the main RESTORE trial | 432 participants (not divided into sub-groups in the post-hoc analysis) | - | sTM | - | Primary outcome: sTM levels correlate with higher 90-day mortality in ventilated paediatric patients (p<0.01)  Secondary outcomes: sTM levels correlate with presence of multi-organ failure (p<0.0001) and worsening oxygenation (p=0.01) but show no association with ventilator free days (p>0.4) or ICU length of stay (p>0.4) |
| Case et al, 2020 ([48](#_ENREF_48)) | Systemic capillary leak syndrome (SCLS) in a 63-year-old man who developed profound shock post-resuscitation | Case report  **Aim:** To highlight profound shock and endothelial dysfunction post-resuscitation in a case of severe acute respiratory syndrome coronavirus 2 (COVID-19) with idiopathic SCLS | Crystalloid volume resuscitation | - | - | - | Systemic capillary leak and impaired microvascular endothelial function | SCLS can occur in acute COVID-19 viral infection and lead to endothelial dysfunction despite administration of 15 litres balanced crystalloid for resuscitation within 24-hours of hospital admission. |
| Bøe et al, 2018 ([49](#_ENREF_49)) | Systemic capillary leak syndrome (SCLS) in a 49-year-old woman with an upper respiratory tract infection who developed profound shock post-resuscitation | Case report  **Aim:** To highlight profound shock and microvascular endothelial damage in a case of SCLS arising from an upper respiratory tract infection | Crystalloid volume resuscitation | - | Syndecan-1 (CD138), and heparan-sulphate | - | - | Administration of 20 litres of intravenous crystalloid for haemodynamic resuscitation led to an increase in syndecan-1 and heparan sulphate markers of endothelial-glycocalyx breakdown and rapid development of generalized peripheral oedema requiring fasciotomies to treat acute compartment syndrome of the lower extremities. Endothelial breakdown markers normalised on disease remission. |
| Somasetia et al, 2014 ([50](#_ENREF_50)) | Endothelial dysfunction in dengue shock syndrome in children aged 2-years to 14-years | Prospective single blind randomised controlled clinical trial conducted between May 2008 and April 2009  **Aim:** To evaluate the effect of hypertonic sodium lactate solution (HSL) on endothelial inflammation | Treatment (hypertonic sodium lactate, HSL) *versus* control (Ringer’s lactate) | 46 participants (divided into 2 groups); HSL (n=24) and Ringer’s lactate (n=22) | - | sVCAM-1 | - | HSL decreased endothelial cell inflammation with significantly lower sVCAM-1 levels at 48-hours (p=0.0024) compared to Ringer’s lactate.  There was similar haemodynamic profiles and recovery from shock in both groups but with lower fluid intake and less accumulation with HSL compared to Ringer’s lactate. |
| *PALICC, Paediatric Acute Lung Injury Consensus Conference; ARDS, acute respiratory distress syndrome; RCT, randomised controlled trial; SOFA, sequential organ failure assessment score; OHCA, out-of-hospital cardiac arrest; GCS, Glasgow coma scale score; ROSC, return of spontaneous circulation; MAP, mean arterial pressure; ATIII, anti-thrombin III; FFP, fresh frozen plasma; EoT, endotheliopathy of trauma; ECVVH, early-initiated continuous venovenous haemofiltration; HES, hydroxyethyl starch; sTM, soluble thrombomodulin; sE-selectin, soluble endothelial leucocyte adhesion molecule; sVEGF, soluble vascular endothelial growth factor; sFLT-1, soluble vascular endothelial growth factor receptor-1; VEcad, vascular endothelial cadherin; sVE-cadherin, soluble vascular endothelial cadherin; vWF, von Willebrand factor; sCD40L, soluble CD40 ligand; tPA, tissue-type plasminogen activator; PAI-1, plasminogen activator inhibitor-1; sVCAM-1, soluble vascular cell adhesion molecule-1; FMD, flow-mediated dilatation; sV-CAM, soluble vascular cell-adhesion molecule; sI-CAM, soluble intercellular adhesion molecule; MFI, microvascular flow index; sP-selectin, soluble platelet adhesion molecule; ANGPT2, angiopoietin 2* | | | | | | | | |

**Supplementary Table S3**: Risk of bias assessment

| **S3(a) Randomised controlled trials (based on Cochrane risk of bias assessment)** | | | | | | | |
| --- | --- | --- | --- | --- | --- | --- | --- |
| **Study** | **Risk of Bias Domains** | | | | | | **Overall risk of bias*** |
|  | 1) Selection bias | 2) Performance bias | 3) Detection bias | 4) Attrition bias | 5) Reporting bias | 6) Other bias or conflict of interest | (score out of 6) |
| Macdonald et al, 2023 ([47](#_ENREF_47)) | - | - | + | - | - | - | ***** (5) |
| Saoraya et al, 2021a ([28](#_ENREF_28)) | - | - | - | ? | - | - | ***** (5) |
| Saoraya et al, 2021b ([34](#_ENREF_34)) | - | - | - | - | - | - | ******(6) |
| Meyer et al, 2020 ([37](#_ENREF_37)) | ? | - | - | - | - | ? | ****(4) |
| Grand et al, 2020 ([38](#_ENREF_38)) | - | - | - | + | ? | - | ****(4) |
| Stensballe et al, 2018 ([24](#_ENREF_24)) | - | - | - | - | - | ? | *****(5) |
| Meng et al, 2016 ([31](#_ENREF_31)) | - | - | + | ? | - | - | ****(4) |
| Turk et al, 2014 ([25](#_ENREF_25)) | ? | ? | ? | - | - | ? | **(2) |
| Somasetia et al, 2014 ([46](#_ENREF_46)) | - | - | - | ? | - | ? | ****(4) |
| Junger et al, 2012 ([27](#_ENREF_27)) | - | - | - | - | - | ? | *****(5) |
| **Key:** *Total score: points awarded based on the number of “-” or low risk of bias  - represents low risk of bias  ? represents unclear risk of bias  + represents high risk of bias | | | | | | | |

| **S3(b) Observational (non-randomised) studies (based on the Newcastle-Ottawa scale for assessment of the risk of bias)** | | | | | | | | |
| --- | --- | --- | --- | --- | --- | --- | --- | --- |
| **Study** | **Risk of Bias Domains** | | | | | | | **Overall risk of bias*** |
|  | **Selection** | | | | **Comparability** | **Outcome** | |  |
|  | 1) Representativeness of the exposed cohort | 2) Selection of the non-exposed cohort | 3) Ascertainment of exposure | 4) Demonstration that outcome of interest was not present at the start of the study | Comparability of exposed and non-exposed cohorts | 1) Assessment of outcome in exposed and non-exposed cohorts | 2) Adequacy of follow-up of exposed and non-exposed cohorts | (score out of 7) |
| Fernández-Sarmiento et al, 2023 ([48](#_ENREF_48)) | ? | - | - | ? | - | - | - | *****(5) |
| Monteiro et al, 2021 ([45](#_ENREF_45)) | - | - | - | ? | - | - | ? | *****(5) |
| Peng et al, 2020 ([18](#_ENREF_18)) | ? | ? | + | - | - | - | ? | ***(3) |
| Lopez et al, 2020 ([19](#_ENREF_19)) | ? | ? | - | ? | - | - | - | *****(4) |
| Welling et al, 2020 ([17](#_ENREF_17)) | - | - | - | ? | - | - | ? | *****(5) |
| Gruen et al, 2020 ([20](#_ENREF_20)) | - | - | - | ? | - | - | - | ******(6) |
| Hippensteel et al, 2019 ([35](#_ENREF_35)) | + | + | - | ? | - | - | - | ****(4) |
| Rovas et al, 2019 ([29](#_ENREF_29)) | - | - | ? | ? | - | - | - | *****(5) |
| Naumann et al, 2019 ([21](#_ENREF_21)) | ? | ? | ? | ? | - | - | - | ***(3) |
| Naumann et al, 2018 ([22](#_ENREF_22)) | ? | ? | ? | ? | - | - | ? | **(2) |
| Gonzalez Rodriguez et al, 2018 ([23](#_ENREF_23)) | - | - | - | - | - | - | - | *******(7) |
| Bourcier et al, 2017 ([30](#_ENREF_30)) | ? | ? | ? | ? | - | - | ? | **(2) |
| Wu et al, 2017 ([36](#_ENREF_36)) | - | - | ? | ? | - | - | ? | ****(4) |
| Ohbe et al, 2017 ([39](#_ENREF_39)) | - | - | ? | ? | - | - | - | *****(5) |
| Bro-Jeppesen et al, 2017 ([40](#_ENREF_40)) | - | - | - | ? | - | - | - | ******(6) |
| Bro-Jeppesen et al, 2016 ([41](#_ENREF_41)) | - | - | - | ? | - | - | - | ******(6) |
| Müller et al, 2016 ([32](#_ENREF_32)) | - | - | - | ? | - | - | - | ******(6) |
| Katundu et al, 2016 ([33](#_ENREF_33)) | ? | ? | ? | ? | - | - | ? | **(2) |
| Tang et al, 2013 ([26](#_ENREF_26)) | - | - | ? | ? | - | - | - | *****(5) |
| Omar et al, 2013 ([42](#_ENREF_42)) | ? | ? | ? | ? | - | - | ? | **(2) |
| **Key:** *Total score: points awarded based on the number of “-” or low risk of bias  - represents low risk of bias  ? represents unclear risk of bias  + represents high risk of bias | | | | | | | | |

**Supplementary Table S4**: Proposed domains for reporting of endotheliopathy in clinical studies

|  | **Characteristics to be described** |
| --- | --- |
| Domain 1 | Quantification of circulating biomarkers of glycocalyx breakdown and shedding in plasma |
| Domain 2 | Quantification of mediators of endothelial function |
| Domain 3 | Quantification of microvascular flow |
